# Optical Inspection of 2D Materials: From Mechanical Exfoliation to Wafer‐Scale Growth and Beyond

**DOI:** 10.1002/advs.202102128

**Published:** 2021-10-29

**Authors:** Yang‐Chun Lee, Sih‐Wei Chang, Shu‐Hsien Chen, Shau‐Liang Chen, Hsuen‐Li Chen

**Affiliations:** ^1^ Department of Materials Science and Engineering National Taiwan University No. 1, Sec. 4, Roosevelt Road Taipei 10617 Taiwan

**Keywords:** 2D materials, enhancing optical signals, optical inspection, van der Waals‐integrated heterostructure, wafer‐scale growth

## Abstract

Optical inspection is a rapid and non‐destructive method for characterizing the properties of two‐dimensional (2D) materials. With the aid of optical inspection, in situ and scalable monitoring of the properties of 2D materials can be implemented industrially to advance the development and progress of 2D material‐based devices toward mass production. This review discusses the optical inspection techniques that are available to characterize various 2D materials, including graphene, transition metal dichalcogenides (TMDCs), hexagonal boron nitride (h‐BN), group‐III monochalcogenides, black phosphorus (BP), and group‐IV monochalcogenides. First, the authors provide an introduction to these 2D materials and the processes commonly used for their fabrication. Then they review several of the important structural properties of 2D materials, and discuss how to characterize them using appropriate optical inspection tools. The authors also describe the challenges and opportunities faced when applying optical inspection to recently developed 2D materials, from mechanically exfoliated to wafer‐scale‐grown 2D materials. Most importantly, the authors summarize the techniques available for largely and precisely enhancing the optical signals from 2D materials. This comprehensive review of the current status and perspective of future trends for optical inspection of the structural properties of 2D materials will facilitate the development of next‐generation 2D material‐based devices.

## Graphical abstract

This review discusses the optical inspection techniques that are available to characterize various 2D materials. The current status and perspective of future trends for optical inspection of the structural properties of 2D materials are comprehensively reviewed, which will facilitate the development of next‐generation 2D material‐based devices.

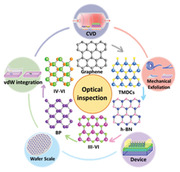

## 1 Introduction

2D materials are a new generation of materials that possess a planar structure having a thickness of only one or a few atoms in one dimension. Thanks to this unique structure, these materials possess novel optical and electrical properties that differ fundamentally from those of their bulk materials; thus, 2D materials have attracted much attention, particularly as promising building blocks for next‐generation photonic and optoelectronic devices,^[^ ^1^ ^]^ including photodetectors,^[^ ^2^ ^]^ optical modulators and switches,^[^ ^3^, ^4^, ^5^ ^]^ light‐emitting devices,^[^ ^6^ ^]^ transistors,^[^ ^7^, ^8^ ^]^ and sensors.^[^ ^9^ ^]^ When applying 2D materials, their basic structural and physical properties are key factors that determine the performance of their resulting devices.^[^ ^10^ ^]^ The layer number and stacking order of the 2D materials,^[^ ^11^ ^]^ the concentrations of structural defects (e.g., crystalline disorders, vacancies, substitutional and interstitial impurities, and edge and grain boundaries) in the 2D materials,^[^ ^12^, ^13^ ^]^ and the types and concentrations of dopants in the 2D materials^[^ ^14^ ^]^ can all significantly influence, for example, their electronic band structures, electrical conductivities, thermal conductivities, and mechanical strengths. Accordingly, there is a need to develop precise techniques for characterizing atomically thin 2D materials at both the material and device level.

Among the many available analytical techniques, optical inspection is a particularly powerful tool for investigating the properties of materials. When the probe light is applied incident to the analytical material (in this case, a 2D material), interactions between the light and the atoms or electrons can reveal much information about the fine structural characteristics. The optical properties of 2D materials have been investigated widely using common optical spectroscopy,^[^ ^15^ ^]^ spectroscopic ellipsometry,^[^ ^16^ ^]^ Raman spectroscopy,^[^ ^17^ ^]^ and photoluminescence (PL) spectroscopy.^[^ ^18^ ^]^ Moreover, analyses of 2D materials performed using single‐photon emission,^[^ ^19^ ^]^ time‐resolved PL, and pump‐probe spectroscopy;^[^ ^20^ ^]^ scattering‐type scanning near‐field optical microscopy (s‐SNOM);^[^ ^21^ ^]^ Fourier transform infrared (FTIR) spectroscopy;^[^ ^22^ ^]^ and X‐ray scattering, diffraction, and reflectivity^[^ ^23^ ^]^ have also allowed a detailed characterization of their structural properties. Optical inspection is a promising method for characterizing 2D materials because of its simplicity and rapid and non‐destructive nature; advances in the field of optical inspection of 2D materials will provide an opportunity for in situ monitoring of the properties of 2D material‐based devices during their fabrication and optimization. In this Review, we summarize the current status—and the challenges and future trends—of the methods available for optical inspection of the characteristics of 2D materials. We provide a comprehensive examination of the techniques for optical inspection of 2D materials with the goal of enhancing progress in the development of novel 2D material‐based devices.

## 2 The Story Begins with Graphene

**Figure 1:**
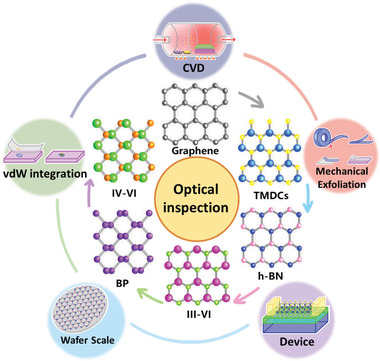
Schematic representation of the most widely studied 2D materials, including graphene, transition metal dichalcogenides (TMDCs), hexagonal boron nitride (h‐BN), group‐III monochalcogenides (III‐VI), black phosphorus (BP), and group‐IV monochalcogenides (IV‐VI), and the developmental progress of 2D materials from mechanical exfoliation to the implementation of 2D material‐based devices.

Since the first successful isolation of monolayer graphene in 2004,^[^ ^24^ ^]^ 2D materials have emerged as wonderful substances with applications in the field of optoelectronics. A 2D material is an atomically thin or layered material having strong in‐plane covalent bonds between its atoms and weak out‐of‐plane van der Waals (vdW) forces between its layers. Graphene, a typical example of a 2D material, was first isolated through mechanical exfoliation, using adhesive tape, from bulk highly oriented pyrolytic graphite (HOPG). This so‐called “Scotch tape method” revolutionized the exploration of many other 2D materials isolated from their bulk crystals,^[^ ^25^ ^]^ allowing examinations of their unique optical and electrical properties. When characterizing the properties of 2D materials, optical inspection provides an efficient method for obtaining structural information in a rapid and non‐destructive manner. As a result, optical inspection is playing an important role in the development of 2D materials (**Figure** **1**); accordingly, there is a need to summarize the current status and future trends of the optical inspection of 2D materials.

Prior to discussing the techniques available for the optical inspection of 2D materials, we briefly review the unique structural, electrical, and optical properties of the most widely studied 2D materials, including graphene, transition metal dichalcogenides (TMDCs), hexagonal boron nitride (h‐BN), group‐III monochalcogenides, black phosphorus (BP), and group‐IV monochalcogenides. In addition, we provide a short summary of the developmental progress of 2D materials, as illustrated in Figure 1. Graphene, the first 2D material to be discovered, comprises a single layer of carbon atoms densely packed into a perfect array of 2D benzene rings. It is a semimetal with a zero‐value bandgap. A universal absorption of 2.3% in graphene has been demonstrated both theoretically (derived by calculating the light absorption of 2D Dirac fermions) and experimentally.^[^ ^15^ ^]^ Furthermore, the Fermi level of graphene can be steadily tuned through electrostatic gating and chemical doping, such that its electrical and optical properties can be controlled precisely.^[^ ^26^ ^]^

Apart from graphene, TMDCs have been the most studied class of 2D materials. They are atomically thin materials having the chemical formula MX_2_, where M is a transition metal and X is a chalcogen. These 2D materials possess several properties that are complementary to those of graphene. For example, TMDCs (e.g., MoS_2_, WS_2_, MoSe_2_, WSe_2_, MoTe_2_) are semiconductors having bandgap energies ranging from 1.0 to 2.5 eV.^[^ ^27^ ^]^ They are indirect bandgap materials when in the form of bulk crystals, but undergo an indirect‐to‐direct transition when the crystals are thinned to monolayers.^[^ ^18^, ^28^ ^]^ In addition, the optical absorption of a monolayer TMDC is relatively strong, reaching a value greater than 10% at two specific peak wavelengths corresponding to the two resonant energies of the TMDC.^[^ ^18^ ^]^ These two optical resonances result from so‐called A and B excitons, respectively.

h‐BN is the most stable crystalline form among the various BN polymorphs. Monolayer h‐BN comprises alternately arranged B and N atoms with strong covalent bonds in a 2D plane, presenting a honeycomb‐like structure analogous to the graphene lattice. Although the crystal structures of h‐BN and graphene are very similar, their electrical properties are quite different. h‐BN is an insulator having an indirect bandgap energy of 5.955 eV.^[^ ^29^ ^]^ Optically, h‐BN is transparent at visible‐IR wavelengths and becomes opaque in the deep ultraviolet (DUV). In addition to its interesting electrical and optical properties, h‐BN has high thermal and chemical stability. Notably, h‐BN possesses an atomically smooth surface without dangling bonds or charge traps; thus, it can be a great supporting substrate or encapsulating material for various 2D material‐based devices.^[^ ^30^ ^]^

Similar to TMDCs, group‐III monochalcogenides are also important 2D semiconductors that have been receiving increasing attention in recent years.^[^ ^31^ ^]^ They feature a layered hexagonal structure having the stoichiometry M_2_X_2_, where M is a group‐III metal (e.g., Ga, In) and X is a chalcogen (e.g., S, Se); each layer comprises two layers of M atoms sandwiched between two layers of X atoms (X–M–M–X). All of the monolayer group‐III monochalcogenides are indirect bandgap semiconductors, with bandgap energies ranging from 2.2 to 3.98 eV.^[^ ^32^ ^]^ Interestingly, some group‐III monochalcogenides—for example, GaSe^[^ ^33^ ^]^ and InSe^[^ ^34^ ^]^—are direct bandgap materials in the bulk crystal and undergo a direct‐to‐indirect transition when thinned to a monolayer, in contrast to the indirect‐to‐direct transition that occurs for monolayer TMDCs. Finally, another important characteristic of group‐III monochalcogenides is that their carrier mobility is higher than that of TMDCs. Such high carrier mobilities make group‐III monochalcogenides competitive candidates for application in high‐speed electronic and optoelectronic devices.

BP, the most thermodynamically stable allotrope of phosphorus, is another single‐element layered material that has been studied widely since its introduction to the 2D material family in 2014.^[^ ^35^ ^]^ BP has a puckered, wavelike structure in which each P atom is connected to three adjacent P atoms, forming a stable orthorhombic crystalline structure. It is a direct bandgap semiconductor having a bandgap energy of 0.3 eV in the bulk crystal, with the bandgap energy increasing upon decreasing the number of layers. When BP is thinned to a monolayer, the bandgap energy increases to approximately 2 eV.^[^ ^36^ ^]^ The most special characteristic of BP is the anisotropic physical properties that arise from its orthorhombic structure, which breaks the three‐fold rotational symmetry.^[^ ^35^ ^]^ The optical properties of BP, including its light absorption and Raman spectra, are all highly anisotropic.

Last, we introduce the group‐IV monochalcogenides, which are isoelectronic analogs of BP having the chemical formula MX, where M is a group‐IV metal (e.g., Sn, Ge) and X is a chalcogen (e.g., S, Se).^[^ ^37^ ^]^ They share a puckered, orthorhombic structure with BP, but in this case, each atomic species is covalently bonded to three neighbors of the other atomic species. Accordingly, a layered structure having zigzag rows of alternating elements is formed. This two‐atom arrangement breaks the inversion symmetry when the group‐IV monochalcogenides are thinned to monolayers; as a result, monolayer group‐IV monochalcogenides possess giant anisotropic piezoelectricity, with piezoelectric coefficients one to two orders of magnitude larger than those of other 2D materials.^[^ ^38^ ^]^ Furthermore, group‐IV monochalcogenides are, in most cases, indirect semiconductors, with the bandgap energy increasing from 1.0 eV in the bulk to 2.3 eV for the monolayer. Finally, the BP‐like structure of group‐IV monochalcogenides suggests that their optical and transport properties should also be highly anisotropic.^[^ ^37^ ^]^

In the early stages of the exploration of 2D materials, mechanical exfoliation was been the most commonly used “top‐down” method for the preparation of monolayers. Because monolayer 2D materials could be isolated directly in the highest quality from single‐crystalline solids when using the “Scotch tape method,” it became possible to study the fundamental properties of these 2D materials. This approach is not, however, ideal for the mass production of 2D materials, and does not always allow precise control over their layer number, lateral size, and crystalline orientation. As a result, chemical vapor deposition (CVD) has been developed as a “bottom‐up” method for the synthesis of monolayer 2D materials of high quality, uniformity, and large area.^[^ ^39^ ^]^ In the CVD process, the formation of the continuous thin film (i.e., the 2D material) can be separated into three different stages: nucleation, growth, and coalescence. Thus, optimization of the growth of 2D materials focuses on tuning these three stages; several parameters (e.g., precursor types, substrates, growing temperature, chamber pressure, carrier gas flow rate, and source‐substrate distance) can significantly influence the structural properties (e.g., layer number, grain size, crystalline orientation, phase, level of doping, and defect density) of 2D materials.^[^ ^39^ ^]^ Therefore, to provide feedback regarding the optimal CVD growth parameters, it would be preferable to have a rapid means of characterizing the structural properties of 2D materials.

In addition to the synthesis of single‐type 2D materials, several researchers have focused on vdW integrations, taking advantage of the establishment of transfer techniques for atomically thin layers.^[^ ^40^ ^]^ The vdW integrations comprise various monolayer or few‐layer 2D materials transferred onto another monolayer or few‐layer 2D material, and so on, with each atomically thin layer integrated with another and stabilized through vdW forces. These structures are particularly interesting for their novel features.^[^ ^41^ ^]^ First, 2D materials can be stacked directly upon others without considering lattice mismatch. Second, no inter‐layer diffusion or segregation of atoms occurs in such structures. Third, the availability of various 2D materials with different properties provides wide flexibility for the design of devices. Most importantly, the combination of different 2D materials in vdW integrations can result in some fascinating properties—for example, interlayer transitions^[^ ^42^ ^]^ and superconductivity^[^ ^43^ ^]^—that differ from the individual intrinsic properties, due to the strong interactions between the pairs of atomic layers. Thus, to monitor the properties of the same 2D materials before and after assembly into vdW integrations with desired properties, it would be ideal to have an accurate and non‐destructive method of characterization.

Recently, a wafer‐scale and single‐crystal h‐BN monolayer was successfully grown on a Cu(111) thin film deposited on a 2 in. *c*‐plane sapphire wafer.^[^ ^44^ ^]^ That study revealed the possibility of growing other wafer‐scale single‐crystal 2D materials, potentially facilitating the widespread industrial application of 2D materials in 2D material‐based devices. Here, a rapid inspection technique would also be useful for characterizing the structural properties of large‐scale 2D materials developed through wafer‐scale growth techniques.

Thus, the developmental progress of various 2D materials (Figure 1) has a common need: a rapid and non‐destructive characterization technique. Optical inspection can fulfill this demand and play a critical role in the fabrication and optimization of 2D materials. Recently, an increasing number of studies into the optical inspection of 2D materials have emerged, with advances in the field potentially paving the way toward future novel 2D materials‐based devices. In the following sections, we provide a comprehensive review of the current status and future trends of the optical inspection of the structural properties of 2D materials.

## 3 Locating 2D materials

Many monolayer and few‐layer 2D materials can be isolated from their bulk crystals by using mechanical exfoliation, the process first used to produce monolayer graphene. Although high‐quality 2D materials can be prepared using this method, it remains a challenge to fabricate 2D material‐based devices from them because it can be difficult to locate such small and ultrathin 2D flakes on substrates when using modern visualization techniques [e.g., atomic force microscopy (AFM), scanning electron microscopy (SEM), transmission electron microscopy (TEM)] because of relatively low throughput. To rapidly locate 2D materials, researchers have developed a visualizing technique based on optical microscopy (OM), which can detect monolayer or few‐layer 2D materials directly when they are prepared on substrates with enhanced visibility.

Investigations into the visibility of 2D materials began with a search for monolayer graphene on the surface of a Si substrate capped with a SiO_2_ layer (SiO_2_/Si), the most commonly used configuration for fabricating field‐effect transistors.^[^ ^45^ ^]^ The visibility of graphene was first described by the optical contrast *C*_r_, defined as the relative intensity of reflected light in the presence (*R*´) and absence (*R*) of graphene:

(1) $$C_{r}=\frac{R-R^{\prime }}{R}$$

**Figure 2:**
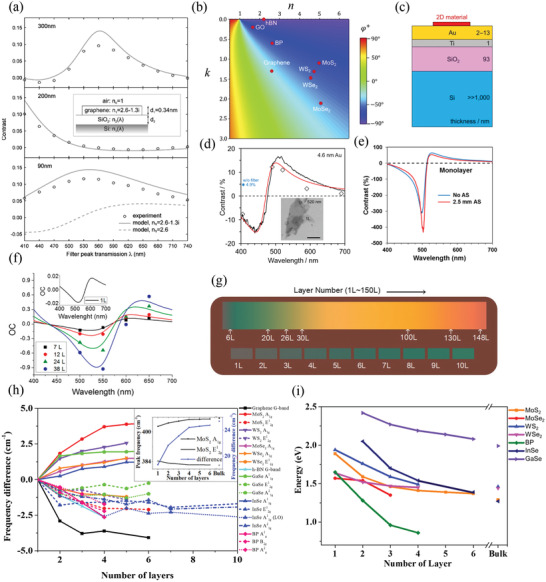
Optimization of optical contrast of 2D materials for location and their layer number‐dependent optical properties. a) Measured (using bandpass filters having a bandwidth of ≈10 nm) and simulated optical contrast of monolayer graphene on SiO_2_/Si substrates having different SiO_2_ thicknesses. Inset: schematic representation of the simulation model. Reproduced with permission.^[^ ^45^ ^]^ Copyright 2007, AIP Publishing. b) Calculated optimal phase map for optical contrast of a thin film. Reproduced with permission.^[^ ^55^ ^]^ Copyright 2019, American Chemical Society. c) Schematic representation of the structure providing optimal optical contrast for 2D materials. d,e) Optimal optical contrast spectra of monolayer graphene oxide (d; red curve: simulated data; black curve: experimental data; diamond markers: experimental data obtained using bandpass filters having a bandwidth of ≈10 nm; inset: optical image of the graphene oxide layer) and monolayer MoS_2_ e) on a 4.6 nm Au film/1 nm Ti/93 nm SiO_2_/Si substrate. c,d) Reproduced with permission.^[^ ^56^ ^]^ Copyright 2019, AIP Publishing. e) Reproduced under the terms and conditions of the CC‐BY license.^[^ ^57^ ^]^ Copyright 2020, The Authors. Published by AIP Publishing. f) Measured (solid symbols) and calculated (curves) optical contrast (OC) of h‐BN having layer numbers from 1 (inset) to 38 on a 290 nm SiO_2_/Si substrate. Reproduced under the terms and conditions of the CC‐BY license.^[^ ^67^ ^]^ Copyright 2019, The Authors. Published by MDPI. g) Simulated colorbar of BP flakes from monolayer (1L, left) to 150 layers (150L, right); the background reveals the color of the substrate. Reproduced with permission.^[^ ^70^ ^]^ Copyright 2019, Wiley‐VCH. h) Raman spectral peak shifts as a function of layer number for Raman spectral peaks measured from various 2D materials; inset: peak positions and peak differences between the A_1g_ and $E_{2g}^{1}$ modes of MoS_2_. i) Measured PL spectral peak energies of various 2D materials plotted with respect to layer number.

Through theoretical and experimental analyses, the optical contrast of graphene was found to depend strongly on both the thickness of the SiO_2_ layer and the wavelength of the incident light. When illuminating through bandpass filters having a bandwidth of approximately 10 nm, the optical contrast at a wavelength of 560 nm could be maximized at 12% for a 300 nm SiO_2_/Si substrate (**Figure** **2**a). Accordingly, a SiO_2_/Si substrate can be an efficient platform for making monolayer graphene and other 2D materials visible.^[^ ^45^ ^]^

In addition to optical contrast, another factor used to quantitatively describe the visibility of 2D materials is the total color difference (TCD).^[^ ^46^ ^]^ The TCD is based on the combination of the reflection spectrum and the International Commission on Illumination (CIE) color space, addressing both the brightness sensitivity and the color perception of the human eye. Using color‐matching functions and the CIE 1976 L*a*b* color space, the effect of the light source and substrate can then be taken into account; the TCD (${\Delta E}_{\mathrm{ab}}^{*}$) between the sample and substrate is calculated using the equation

(2) $$\Delta E_{\mathrm{ab}}^{*}=\sqrt{{\left(\Delta L^{*}\right)}^{2}+{\left(\Delta a^{*}\right)}^{2}+\;{\left(\Delta b^{*}\right)}^{2}}$$

where ∆L* is the lightness difference (representing the contrast without the effect of color factors) and ∆a* and ∆b* are the color difference. According to the National Bureau of Standards, an image can be distinguished well if the TCD is larger than 1.5. Notably, the larger the value, the more readily we can distinguish the color difference.^[^ ^46^ ^]^

The visibilities of monolayer graphene and TMDCs have been optimized through maximizing their optical contrasts or TCDs on various kinds of substrates.^[^ ^46^, ^47^, ^48^, ^49^, ^50^ ^]^ Although Al_2_O_3_/Si^[^ ^46^ ^]^ and Si_3_N_4_/Si^[^ ^49^, ^50^ ^]^ substrates provide better visibility for both graphene and TMDCs, the SiO_2_/Si substrate, especially the standard 300 nm SiO_2_/Si, remains the structure used most often for visualizing many other monolayer 2D materials, including h‐BN,^[^ ^51^ ^]^ InSe,^[^ ^52^ ^]^ BP,^[^ ^53^ ^]^ and GeSe.^[^ ^54^ ^]^ When using the 300 nm SiO_2_/Si substrate, it is possible to rapidly locate and investigate the in‐plane optical anisotropy of BP and GeSe, including their anisotropic light absorption, reflection, refractive index, and extinction coefficient, thanks to the acceptable optical contrast.^[^ ^53^, ^54^ ^]^ It remains difficult, however, to use the human eye to visualize the optical images of some transparent 2D materials on 300 nm SiO_2_/Si. For example, monolayer h‐BN has a low white‐light contrast of less than 1.5%; a maximized contrast of only approximately 2.5%, similar to that of graphene on a glass substrate in light‐transmission mode, has been achieved when using a thinner SiO_2_ layer (≈ 80 ± 10 nm).^[^ ^51^ ^]^ To determine the optimal substrate that provides the highest contrast for h‐BN, as well as those for other 2D materials, a comprehensive investigation of the optical contrast was conducted theoretically in terms of the effects of the optical properties of 2D materials, the substrates, and the light illumination conditions.^[^ ^55^ ^]^ By considering the role of the substrate to be represented as a single complex reflectivity, regardless of the structural details, high optical contrast can be achieved when the original reflection of the substrate is sufficiently low. In addition, each 2D material has its own specific reflectivity phase in which it achieves high optical contrast, determined by its distinct optical constants. Figure 2b displays the calculated optimal phase map (*φ* ^+^) as functions of the refractive index (*n*) and extinction coefficient (*k*) of a thin film; the optimal phases of various 2D materials are highlighted.^[^ ^55^ ^]^ According to this map, the optimal phase for any 2D material can be found when its optical constants are known. Furthermore, the light illumination conditions are an important factor when improving the optical contrast, because light arrives incident from a range of angles in an OM system having an objective lens of high numerical aperture (NA). The reflectances at various incident angles and with different degrees of polarization, as well as the angular distribution of the light power, should be taken into account for optimization. Based on such a study, common criteria can be established when designing substrates of high optical contrast for a given 2D material.^[^ ^55^ ^]^

Following the criteria mentioned above, substrates that provide high optical contrast for graphene oxide (GO)^[^ ^56^ ^]^ and MoS_2_^[^ ^57^ ^]^ were developed by introducing a thin gold (Au) film (<13 nm) between the 2D material and the SiO_2_/Si substrate (Figure 2c). The introduction of the Au film adjusted the reflectivity phase of the substrate to ensure the optimal phase for these 2D materials. By optimizing the thickness of the Au film and considering the different NAs of the objective lens, the optical contrast for monolayer GO was enhanced to approximately 17% when using a 50× objective (Figure 2d);^[^ ^56^ ^]^ furthermore, the optical contrast for monolayer MoS_2_ was enhanced significantly to an extremely high value of 430% when using a 10 × objective with a 2.5 mm aperture stop (AS) (Figure 2e).^[^ ^57^ ^]^ The enhanced optical contrast on the optimal substrates suggests that even very small flakes of a 2D material can be readily located. Notably, because the optical constants of GO are close to those of h‐BN, the optimal substrate for visualizing monolayer h‐BN should be identified in the near future.^[^ ^55^ ^]^

So far, we have reviewed the visualizing techniques to detect 2D materials using the OM in reflection mode. Another technique based on instruments similar to conventional OM but focusing the thermal radiation has also been developed for visualizing 2D materials, which is called “radiation‐mode OM.“^[^ ^58^ ^]^ The radiation‐mode OM have been used to establish the in situ and real‐time observation on the growth of graphene on Cu during the CVD process.^[^ ^58^, ^59^ ^]^ At high temperature, both the thermal radiations from the graphene and the Cu substrate are strong so that sufficient contrast could be obtained depending on the difference in their emissivities. Using this technique, the growth mechanism of CVD graphene can be observed and the growing parameters can be optimized. We believed that such technique could also be very suitable for establishing the in situ and real‐time monitoring during the growth of other 2D materials.

## 4 Identifying the Layer Number of 2D Materials

Quantum confinement effects in the direction perpendicular to the plane of a 2D material result in several unique thermal, optical, electrical, and mechanical properties when it reaches an atomically thin layer; accordingly, these properties are strongly related to the layer number of a 2D material. When applying 2D materials, controlling and identifying their actual layer numbers will be a very important issue to ensure the required performance of 2D material‐based devices. As mentioned above, AFM and TEM have been used to locate 2D materials and to count their layer numbers with high spatial resolution;^[^ ^60^, ^61^ ^]^ these methods are, however, time‐consuming and require relatively complex processes for sample preparation. Moreover, irradiation of the electron beam during TEM inspection can result in unexpected sample damage. To avoid such problems, several optical methods have been developed to identify the layer numbers of 2D materials.

The intuitive optical method for identifying the layer numbers of 2D materials would be to measure their transmittance, reflectance, and absorptance spectra and then investigate the relationship between these optical characteristics and the layer number.^[^ ^15,62^ ^]^ Nevertheless, the layer number‐dependent optical spectroscopy of 2D materials requires careful consideration of the effect of the substrate, which can significantly influence the characterization of 2D materials.^[^ ^63^ ^]^

Considering the substrate effect, a common strategy for counting the layer number is to transfer the 2D material onto a substrate that provides high optical contrast. The high optical contrast of the 2D materials on those substrates means that the interaction between the incident light and the 2D materials is strong; therefore, the differences in the optical signals between pairs of layer numbers can be increased, thereby allowing the ready distinction of the layer numbers of the 2D materials. To date, the optical contrast spectra as a function of layer number have been experimentally and theoretically investigated for mechanically exfoliated graphene (1 to 10 layers),^[^ ^64^ ^]^ TMDCs (1 to 13 layers),^[^ ^65^, ^66^ ^]^ h‐BN (1 to 50 layers),^[^ ^67^ ^]^ GaS and GaSe (1 to 10 layers),^[^ ^68^ ^]^ InSe (1 to 5 layers),^[^ ^69^ ^]^ and BP (1 to 7 layers)^[^ ^70^ ^]^ on various substrates, including SiO_2_/Si,^[^ ^64^, ^65^, ^66^, ^67^, ^68^, ^69^, ^70^ ^]^ Al_2_O_3_/Si,^[^ ^70^ ^]^ and Si_3_N_4_/Si.^[^ ^70^ ^]^ Notably, the optical contrast per monolayer of h‐BN on 290 nm SiO_2_/Si is less than 2% (Figure 2f),^[^ ^70^ ^]^ but it can be increased by optimizing the optical contrast of the substrate.^[^ ^55^ ^]^ Although the layer numbers of various 2D materials can be identified directly by using the peak value of the optical contrast, this method relies on a spectrometer to obtain the optical contrast spectra; therefore, it is time‐consuming and not suitable for large‐scale samples. As a result, a simpler and faster identification technique—optical imaging—has been developed to identify the layer numbers of 2D materials.

The optical imaging technique also involves transferring a 2D material onto a substrate that provides high optical contrast (e.g., standard 300 nm SiO_2_/Si), but identification of the layer number is performed by visualizing OM images, without using a spectrometer. The layer numbers of 2D materials can be identified by comparing the color between the sample and a reference color.^[^ ^46^, ^70^, ^71^ ^]^ The color discussed here is described by the standard RGB (sRGB) values, based on consideration of the spectral power distribution of the incident light, the light illumination conditions, the reflectance spectrum of the sample, and the International Commission on Illumination (CIE) color space. Meanwhile, the color corrections of the OM system, resulting from the image captured by the objective lens and visualized by the eyepieces or display, should also be considered. Using experimental sRGB values determined from a sample of a 2D material of known structure, these parameters can be suitably adjusted and the impact of OM system offset; therefore, the reference sRGB values can be established and, subsequently, so can the colors of the sample as a function of the layer number. Several studies have applied this color‐based method to rapidly determine the layer numbers of graphene,^[^ ^46^ ^]^ h‐BN,^[^ ^71^ ^]^ and BP.^[^ ^70^ ^]^ In particular, the establishment of reference colors provides a very convenient way of roughly determining the layer numbers of 2D materials directly with the naked eye. Figure 2g provides an example of a theoretically simulated colorbar of BP flakes having various layer numbers.^[^ ^70^ ^]^ Notably, because the color difference depends on several experimental parameters and the nature of the recording system, it can be difficult to find a specific solution (i.e., colorbar) that works for all systems.^[^ ^72^ ^]^

So far, we have reviewed methods for identification of the layer numbers of 2D materials using optical spectroscopy, optical contrast, and optical imaging techniques. Raman spectroscopy is another powerful optical inspection tool for identifying the layer numbers of 2D materials, because many Raman spectral features of 2D materials are strongly dependent on the layer number, due to interlayer interactions. For example, the main Raman peaks of graphene are the G band (ca. 1580 cm^‐1^), which corresponds to the in‐plane E_2g_ vibration of the carbon atom pairs, and the second‐order 2D band (ca. 2700 cm^‐1^). The G band downshifts upon increasing the layer number, and the 2D band undergoes splitting as a function of layer number, resulting in various shapes.^[^ ^73^, ^74^ ^]^ In the case of TMDCs, the out‐of‐plane A_1g_ vibration mode stiffens (upshifts) while the in‐plane $E_{2g}^{1}$ vibration mode softens (downshifts) upon increasing the layer number.^[^ ^75^, ^76^, ^77^ ^]^ Furthermore, the main Raman spectral peaks of h‐BN (G band),^[^ ^78^ ^]^ GaSe ($A_{1g}^{1}$, $E_{2g}^{1}$, and $A_{1g}^{2}$),^[^ ^79^ ^]^ InSe [$A_{1g}^{1}$, $E_{2g}^{1}$, $A_{1g}^{1}$(LO), and $A_{1g}^{2}$],^[^ ^79^ ^]^ BP ($A_{g}^{1}$, B_2g_, and $A_{g}^{2}$),^[^ ^80^ ^]^ and several group‐IV monochalcogenides, including SnS, SnSe, GeS, and GeSe (B_1u_, B_2g_, $A_{g}^{2}$, and $E_{3g}^{2}$),^[^ ^81^ ^]^ also undergo an upshift or downshift upon increasing the number of layers from a monolayer to a few layers. Figure 2h summarizes the Raman spectral peak shifts with respect to the layer number for various 2D materials. The Raman spectral peak positions of these monolayer 2D materials are also displayed and set as their respective references. Here, we do not include the group‐IV monochalcogenides because their Raman spectral peak shifts have been investigated only theoretically.^[^ ^81^ ^]^ As revealed in Figure 2h, upon increasing the layer number, all of these Raman spectral peaks shift by only approximately 1–4 cm^‐1^. For easier identification, the peak differences between the A_1g_ and $E_{2g}^{1}$ modes of TMDCs^[^ ^75^, ^76^ ^]^ and between the $A_{1g}^{1}$ and $A_{1g}^{2}$ modes of GaSe^[^ ^79^ ^]^ and InSe,^[^ ^79^ ^]^ respectively, have been used as a more obviously differentiated “layer number indicator” because these modes shift in opposite directions upon increasing the layer number. The inset to Figure 2h presents an example of the peak difference between the A_1g_ and $E_{2g}^{1}$ modes of MoS_2_. Notably, MoSe_2_ provides only one A_1g_ peak when the excitation energy is less than 2.54 eV (corresponding to 488 nm);^[^ ^82^ ^]^ on the other hand, the A_1g_ and $E_{2g}^{1}$ modes of WSe_2_ are not fully separated.^[^ ^77^ ^]^ Accordingly, the peak differences for these TMDCs are not as good indicators when compared with those for MoS_2_.

The intensity of the Raman spectral signals of 2D materials can also be used to identify their layer number. Indeed, the ratio of the integrated intensities of the graphene G and 2D bands,^[^ ^73^ ^]^ the intensity ratio of the MoS_2_ A_1g_ and $E_{2g}^{1}$ modes relative to that of the Si substrate (≈520 cm^‐1^),^[^ ^83^ ^]^ and the peak intensities of the main Raman spectral peaks of h‐BN (G band)^[^ ^78^ ^]^ and BP ($A_{g}^{1}$, B_2g_, and $A_{g}^{2}$)^[^ ^80^ ^]^ all increase upon increasing the layer number. Moreover, the intensity of the Si peak from the conventional SiO_2_/Si substrate decreased upon increasing the layer numbers of such 2D materials as graphene,^[^ ^84^ ^]^ MoS_2_,^[^ ^85^ ^]^ and WSe_2_.^[^ ^85^ ^]^ Although these Raman spectral peak intensities can be used as an indicator of the number of layers, the intensity of a Raman spectral peak also depends on several other parameters, including the thickness of the SiO_2_ layer of the SiO_2_/Si substrate,^[^ ^86^, ^87^ ^]^ the focusing condition of the objective lens in the Raman spectrometer, and the polarization‐dependent Raman spectral intensity arising from anisotropic 2D materials (e.g., the $A_{g}^{1}$ mode of BP).^[^ ^88^ ^]^ Therefore, calibration of the Raman spectral intensity should be performed carefully by measuring the Raman spectra of 2D materials of known structure, prior to counting their layer numbers.

Because the electronic band structures of semiconducting 2D materials vary with respect to the number of layers, analysis of their PL spectra can provide information about their layer numbers. Figure 2i summarizes the measured PL peak energies of TMDCs,^[^ ^18^, ^89^, ^90^ ^]^ InSe,^[^ ^91^ ^]^ GaSe,^[^ ^92^ ^]^ and BP^[^ ^93^ ^]^ with respect to the layer number. Notably, in the case of TMDCs, the PL spectral peak energies are determined from the lowest‐energy peak in the PL spectra, corresponding to the bandgap transition energy (usually named the “A peak” for the A exciton peak when the layer number is equal to one and the “I peak” for the indirect bandgap transition when the layer number is greater than or equal to two). In addition to the peak energy, the PL spectral peak intensities of TMDCs,^[^ ^18^, ^89^, ^90^ ^]^ InSe,^[^ ^91^ ^]^ and GaSe^[^ ^92^ ^]^ are other signatures for distinguishing their layer numbers, because TMDCs undergo an indirect‐to‐direct transition, while InSe and GaSe undergo direct‐to‐indirect transitions, when they are thinned to a monolayer. The PL quantum yields and PL spectral intensities of TMDCs (InSe and GaSe) increase (decrease) significantly upon decreasing the layer number.

X‐ray spectroscopies, such as X‐ray scattering, diffraction, and reflectivity, are typical techniques used to study the crystalline structure of materials (e.g., lattice spacings, which will be discussed later) and the thickness of thin film by investigating the interactions between X‐rays [having wavelengths in the range from 0.5 to 2 Å] and the periodically arranged atoms (is of the order of a few Å). For 2D materials, several investigations related to determining their thickness (in other words, the layer numbers) using X‐rays based techniques have also been reported. For example, the layer number of multilayer graphene was identified by analyzing the relationship between the thickness and the full width at half maximum (FWHM) of the (002) peak in their X‐ray diffraction spectra.^[^ ^94^ ^]^ The structural properties including the thickness and surface roughness of graphene,^[^ ^95^ ^]^ WS_2_,^[^ ^96^ ^]^ and h‐BN^[^ ^97^ ^]^ were probed by investigating the X‐ray reflectivity. Although X‐rays based techniques are useful tools for identifying the layer number of 2D materials, fitting procedures are still needed; therefore, it must be noted that the fitting parameters should be carefully addressed to obtain the correct results when using such techniques.

## 5 Identifying Defects in 2D Materials

2D materials always contain inevitable intrinsic defects that can influence their properties significantly. Various type of defects, including vacancies, crystalline disorder, adatoms, substitutional impurities, grain boundaries, and edges, have been observed in 2D materials.^[^ ^98^ ^]^ These defects can behave as carrier donors and scattering, trap, and recombination centers; thus, carrier transport would be hindered by such defects, resulting in low carrier mobilities and prolonged response times for 2D material‐based devices, while photoexcited electron/hole pairs (i.e., excitons) in 2D materials would undergo non‐radiative recombination at the defect site, lowering their quantum yields for light‐emitting applications. On the other hand, the presence of certain defects in 2D materials, especially vacancies, crystalline disorders, and grain boundaries, can affect the mechanical strength, depending on the atomic arrangement of these defects. Furthermore, defects can also interact with thermal phonons in 2D materials, thereby disrupting the transport of thermal energy and resulting in lower thermal conductivity. Because both the type and number of defects can affect the electrical, optical, mechanical, and thermal properties of 2D materials,^[^ ^98^ ^]^ non‐destructively identifying and quantifying defects in 2D materials should be a crucial step prior to their application in various devices.

**Figure 3:**
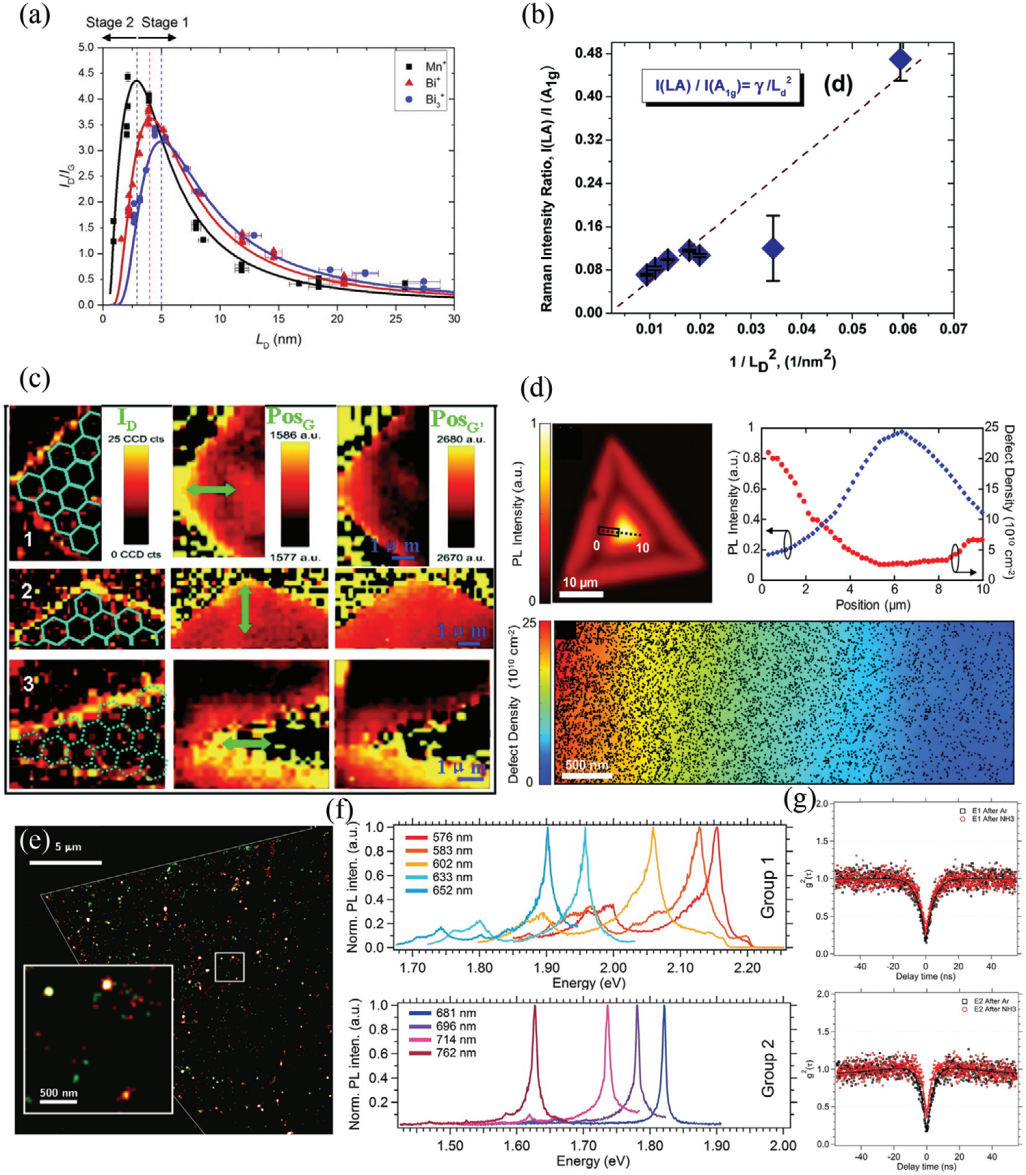
Defect‐related optical properties of 2D materials. a) Intensity of graphene D band (*I*_D_) relative to that of G band (*I*_G_), plotted with respect to the average distance between defects (*L*_D_); the fitted curves are also displayed. Reproduced with permission.^[^ ^99^ ^]^ Copyright 2014, AIP Publishing. b) Correlation between the intensity of the LA(M) mode relative to that of the A_1g_ mode and the value of 1/$L_{D}^{2}$ of monolayer MoS_2_. Reproduced under the terms and conditions of the CC‐BY license.^[^ ^105^ ^]^ Copyright 2020, The Authors. Published by The Royal Society of Chemistry. c) Raman mapping of the D band intensity (left), G band peak position (middle), and 2D band (also denoted as G´ band) peak position (right) obtained from monolayer graphene using polarization‐dependent Raman spectroscopy; green arrow indicates the direction of polarization of incident laser light. Reproduced with permission.^[^ ^112^ ^]^ Copyright 2010, American Chemical Society. d) PL characteristics of monolayer WS_2_ on a polydimethylsiloxane (PDMS) substrate. Upper left: mapping of PL intensity; bottom: mapping of defect positions (black dots) measured from the rectangle regions indicated in the PL mapping, performed using conductive atomic force microscopy (CAFM); upper right: PL intensity and correlated defect density plotted with respect to the position obtained from the dashed line in the PL mapping. Reproduced with permission.^[^ ^119^ ^]^ Copyright 2018, American Chemical Society. e) PL image of defective multilayer h‐BN. Reproduced with permission.^[^ ^127^ ^]^ Copyright 2019, American Chemical Society. f) Normalized spectra of the single‐photon emissions (SPEs) measured from defective h‐BN. Top: group 1; bottom: group 2. g) Measured second‐order autocorrelation functions g^2^(*τ*) from the group 1 (E1, top) and group 2 (E2, bottom) defects after 30 min of annealing under an Ar or NH_3_ environment. f,g) Reproduced with permission.^[^ ^126^ ^]^ Copyright 2016, American Chemical Society.

Raman spectroscopy has been used widely for many years to investigate defects in 2D materials. When defects are present in 2D materials, the lattice vibrations would be disturbed, thereby changing the Raman spectra. These changes in Raman spectra have been studied by intentionally introducing defects into 2D materials through ion bombardment,^[^ ^99^ ^]^ electron beam irradiation,^[^ ^100^ ^]^ chemical reactions (e.g., oxidation, fluorination),^[^ ^101^ ^]^ and plasma treatment,^[^ ^101^ ^]^ such that relationships between the defect‐related Raman spectral responses and the nature and number of defects can be established and regarded as references for identifying unknown defects. Typically, the most pronounced defect‐related Raman spectral response for a 2D material is the appearance of new defect‐activated Raman spectral peak that would be forbidden in a perfect 2D crystal; its intensity can be used to determine the defect density. For example, when defects are introduced into graphene, defect‐activated D and D´ bands (located near 1350 and 1600 cm^‐1^, respectively) appear in its Raman spectrum.^[^ ^101^ ^]^ The intensity of the D band (*I*_D_) relative to that of the G band (*I*_G_) is strongly related to the average distance between the defects (*L*_D_) and the size of the defects.^[^ ^102^ ^]^ This relationship has been studied by deliberately creating defects through the bombardment of 25 keV Mn^+^, Bi^+^, and Bi^3+^ ions, resulting in defect sizes of 0.6 ± 0.1, 1.3 ± 0.1, and 1.9 ± 0.1 nm, respectively (**Figure** **3**a).^[^ ^99^ ^]^ For a given defect size, the *I*_D_/*I*_G_ ratio increased upon increasing the defect density (i.e., *L*_D_ decreased) and then reached a maximum that corresponded to the overlap of the Raman‐activated regions around the defects. The values of *L*_D_ of such maxima divided the relationship into regions of low (stage 1) and high (stage 2) defect densities. When the defect size increased, the *I*_D_/*I*_G_ ratio at low defect density (within stage 1) increased. Accordingly, one cannot accurately determine the defect density from the *I*_D_/*I*_G_ ratio alone without knowing the size of the defect. Thus, the graphene defect size should also be obtained separately to quantify the defects in terms of the graphene *I*_D_/*I*_G_ ratio in the Raman spectrum.

In addition to using the *I*_D_/*I*_G_ ratio to estimate the defect density, the intensity of the D´ band relative to that of G band (*I*_D´_/*I*_G_) can also be established because there is a linear dependence between these two parameters, even though the intensity of the graphene D´ band is usually low.^[^ ^101^ ^]^ More importantly, the *I*_D_/*I*_D´_ ratio in graphene is distinctive for various types of defects, including sp^3^‐hybridized defects (*≈*13),^[^ ^101^ ^]^ vacancy‐like defects (*ca*. 7),^[^ ^101^ ^]^ boundaries (*ca*. 3),^[^ ^101^ ^]^ and B‐ and N‐substitutional defects (≈2);^[^ ^103^ ^]^ thus, this information can reveal the nature of the defect in a graphene sample. Notably, the *I*_D_/*I*_D´_ ratios of the various types of defects were only determined in this specific case; they should be predetermined when establishing such a Raman spectral inspection method to identify the types of defects in other samples.

In the case of monolayer MoS_2_, a new defect‐activated Raman spectral peak, assigned as the LA(M) mode near 227 cm^‐1^, appeared after defects were introduced.^[^ ^104^, ^105^ ^]^ Interestingly, the intensity of the signal for the LA(M) mode relative to that of the A_1g_ or $E_{2g}^{1}$ mode behaved analogously to the *I*_D_/*I*_G_ ratio in graphene, considered an indicator of the defect density (Figure 3b),^[^ ^105^ ^]^ and is described as follows:

(3) $$\frac{I(\mathrm{LA})}{I(A_{1g}\;\mathrm{or}\;E_{2g}^{1})}=\frac{\gamma }{L_{D}^{2}}$$

where *γ* is the correlation constant. Although *γ* was reported to be dependent on the laser excitation energy used in the Raman spectral system, different *γ* values (ranging from 0.59 to 7.5 nm^2^) had been obtained at the same laser excitation energy of 2.3 eV (532 nm) in previous research;^[^ ^104^, ^105^ ^]^ thus, the correlation constant *γ* might depend on other parameters that have yet to be studied. We suspect that the nature of the defects influenced the value of *γ*; therefore, it could possibly be used to identify the defects in 2D materials.

Other defect‐related Raman spectral responses for 2D materials, including positional shifts, broadening, and changes in intensity (or intensity ratio) of their prominent Raman spectral peaks, can also be used to identify defect densities in 2D materials. The FWHM of the three main Raman spectral peaks for graphene (D, G, and 2D bands) increase linearly upon increasing the defect density, due to the scattering of phonons induced by defects.^[^ ^106^ ^]^ On the other hand, when the defect is introduced into TMDCs, the A_1g_ mode of monolayer MoS_2_ upshifts and broadens asymmetrically toward higher frequency, while the $E_{2g}^{1}$ mode downshifts and asymmetrically broadens toward lower frequency;^[^ ^104^, ^105^ ^]^ both the A_1g_ and $E_{2g}^{1}$ modes of monolayer WS_2_ downshift and broaden toward lower frequency;^[^ ^107^ ^]^ the A_1g_ mode of MoSe_2_ downshifts and broadens toward lower frequency,^[^ ^108^ ^]^ while the A_1g_ mode of WSe_2_ displays the opposite behavior, with an upshift and broadening toward lower frequency;^[^ ^107^ ^]^ the $E_{2g}^{1}$ mode of MoTe_2_ does not shift significantly, while its FWHM increases in the presence of defect.^[^ ^109^ ^]^ Furthermore, because BP is sensitive to O_2_ and humidity, monolayer or few‐layer BP degrades rapidly in an ambient environment, with the Raman spectral intensity of the $A_{g}^{1}$ mode relative to that of the $A_{g}^{2}$ mode decreasing upon increasing the oxidation time;^[^ ^110^ ^]^ this ratio can also be used as an indicator of defect density in BP.

Using Raman spectroscopy, one can identify the types and number of defects in various 2D materials through investigating their defect‐related Raman spectral responses. Moreover, the edge chirality of 2D materials can be characterized using polarization‐dependent Raman spectroscopy.^[^ ^111^, ^112^, ^113^, ^114^ ^]^ For example, when polarized laser light is applied incident to monolayer graphene edges of different chirality (zigzag and armchair) at the same angle, the intensity of the D band of the armchair edge is stronger than that of the zigzag edge; meanwhile, both the G and 2D bands upshift (downshift) at the zigzag (armchair) edge (Figure 3c).^[^ ^112^ ^]^ Accordingly, the edge chirality and, hence, the crystal orientation of graphene can be identified readily. Furthermore, when polarized laser light is applied incident to such anisotropic 2D materials as BP,^[^ ^113^ ^]^ GeS,^[^ ^114^ ^]^ and GeSe^[^ ^114^ ^]^ at various polarization angles, their Raman spectra are polarization‐dependent and can be analyzed using a polarizer. Under certain polarization configurations (crossed or parallel to the incident polarization), some Raman‐forbidden modes can be observed at the zigzag (armchair) edge while vanishing at the armchair (zigzag) edge, the result of the distortion of the incident electromagnetic field; thus, these Raman‐forbidden modes can be characteristic Raman spectral modes for the identification of edge chirality. To date, however, they have been observed only for flakes of greater thickness; there might be a need to enhance the Raman spectral scattering signals for observation of the edge Raman modes in monolayers and, thereby, identify their edge chiralities. The enhancing techniques for Raman spectroscopy are discussed later.

It is also worth noting that edge chirality is an important structural characteristic in 2D material nanoribbon or quantum dots which would significantly influence their electrical and thermal properties. For example, the zigzag edge of graphene was found to be metallic while the armchair edge was found to be semiconducting;^[^ ^115^ ^]^ therefore, the graphene nanoribbon with higher fraction of zigzag edges exhibits smaller bandgap than the armchair‐edge nanoribbon of similar width. That is, the bandgap of graphene nanoribbon is strongly dependent on their edge chirality. In the case of BP nanoribbon, the thermal conductivity was found to be largely reduced at the armchair edges due to the phonon boundary scattering, making armchair‐edge BP nanoribbon more suitable for thermoelectric applications.^[^ ^116^ ^]^ According to these studies, the edge chirality indeed play a key role in electric and thermoelectric applications of 2D material nanoribbon and should be accurately identified during the optimization of their preparation process so that the desired properties could be obtained.

Defects in 2D materials can interact with free charge carriers and excitons, significantly influencing their recombination during PL; therefore, the PL spectra of 2D materials could possibly provide information regarding the nature and number of defects. Taking TMDCs as an example, the PL spectra of monolayer TMDCs are dominated by the signal for their free neutral exciton emission (A exciton), labeled as X_0_. Asymmetric broadening of this main PL feature toward lower energy is often observed, corresponding to the charged exciton emission (or trion emission) having a lower radiative quantum efficiency, labeled as X^‐^. This signal can be attributed to the binding of neutral excitons to excess electrons, originating from the n‐type doping arising from the presence of intrinsic chalcogenide vacancies in TMDCs. Moreover, the defect‐bound exciton emission (labeled as X_B_), located approximately 0.1 eV below the bandgap, can also be found, the result of localization of neutral excitons by defects. Overall, the relationship among the peak intensities of the free (X_0_), charged (X^‐^), and bound (X_B_) exciton emissions (note that these peaks are located at different energies) can be an indicator of the defect density in monolayer TMDCs.^[^ ^117^, ^118^ ^]^ In general, a lower intensity for the neutral emission and a higher intensity for the emission of the charged or bound exciton corresponds to a higher number of defects;^[^ ^119^, ^120^ ^]^ this correlation has been applied to monitor structural quality during optimization of the growth of monolayer TMDCs (Figure 3d).^[^ ^120^ ^]^

When investigating defects in 2D materials through PL spectroscopy, a higher intensity for the neutral exciton emission (X_0_) and a lower intensity for the defect emission (X^‐^ or X_B_) usually implies a lower number of defects, but this correlation is not always valid—for example, in the case of TMDCs, where the defects usually feature several dangling bonds that function as active sites for molecular adsorption. In this case, N_2_,^[^ ^117^ ^]^ O_2_,^[^ ^121^, ^122^ ^]^ and H_2_O^[^ ^122^ ^]^ gas molecules can readily adsorb onto the defect sites physically or chemically, introducing p‐type doping that can interact with negatively charged trions to form stable excitons. Accordingly, a conversion from trion (X^‐^) to exciton (X_0_) emission occurs in the presence of adsorbed gas molecules on defects, such that the intensity of the X_0_ peak is increased. That is, good optical quality (high PL peak intensity and narrow FWHM) does not necessarily imply good crystal quality.^[^ ^117^ ^]^ Such adsorption of gas molecules should be taken into account when using PL spectroscopy to characterize 2D materials, and it is suggested that some other optical inspection techniques (e.g., Raman spectroscopy) should be applied associatively.

Defects can also create energy levels within the bandgap of h‐BN, but they play a different role when compared with those in semiconducting 2D materials (e.g., TMDCs and BP). Here, we review the emission properties of defects in h‐BN and discuss how they can be applied with optical inspection techniques to probe defects. To date, the emissions from defects in monolayer or multilayer h‐BN have mostly been probed using sub‐band‐gap excitation laser light.^[^ ^123^, ^124^, ^125^, ^126^, ^127^ ^]^ Under normal circumstances, the sub‐band‐gap energy of the laser light cannot excite electron/hole pairs in h‐BN, which possesses a bandgap energy of approximately 6 eV;^[^ ^29^ ^]^ when defects are present in h‐BN, however, various defect levels arise within the bandgap and some of them can be treated as ground and excited states of an isolated emitter, allowing carriers to be excited by low‐energy lasers (even laser light at visible wavelengths). Accordingly, only defect sites can emit light, and they can be identified by directly visualizing the bright spots in the PL map. Hence, the defect density of h‐BN can be determined simply by counting the number of such bright spots. Figure 3e provides an example of the PL image of a defective multilayer h‐BN excited by a 561 nm laser.^[^ ^127^ ^]^

An interesting characteristic can be observed in this PL image: there exist at least two types of bright spots, exhibiting different colors. It is believed that these distinctive emissions originate from different types of defects. If the correlation between the emission properties and the defect structures could be identified, it would be possible to characterize the nature of the defects in h‐BN samples. The spectral responses from defects in monolayer and multilayer h‐BN have been investigated widely, and several defect‐related PL spectral emission peaks have been reported, locating near 5.5 eV (≈227 nm),^[^ ^123^ ^]^ 4.1 eV (≈ 300 nm),^[^ ^123^, ^124^ ^]^ and 2 eV (≈623 nm).^[^ ^125^, ^126^ ^]^ After excitation by visible‐wavelength lasers, the emissions located near 2 eV (indeed, in the energy range from 1.63 to 2.16 eV, corresponding to the wavelength range from 575 to 762 nm) have been characterized as single‐photon emissions (SPEs) at room temperature (Figure 3f);^[^ ^126^ ^]^ this phenomenon can be verified by measuring the second‐order autocorrelation functions g^2^(*τ*), using a Hanbury‐Brown and Twiss (HBT) interferometry setup (Figure 3g).^[^ ^126^ ^]^ SPE is a very special characteristic associated with defective emissions, and it has been received great attention.^[^ ^125^, ^126^, ^128^, ^129^, ^130^ ^]^ An ideal SPE is a phenomenon in which only a single photon is emitted at a time from a light source. Such light sources, called “single‐photon emitters” or “quantum emitters,” are significant for use in a variety of quantum technologies. SPE has been observed from the defect states of various 2D materials, including WSe_2_,^[^ ^19^, ^131^ ^]^ MoS_2_,^[^ ^132^ ^]^ GaSe,^[^ ^133^ ^]^ and h‐BN.^[^ ^125^, ^126^ ^]^ It can be observed from h‐BN, however, only at room temperature, because the defect levels involved with SPE in h‐BN are generally far from the valence and conduction bands (hence, they are called “deep levels”); thus, these defect levels are highly isolated, and the relaxation of photoexcited carriers usually does not involve thermal phonons. Accordingly, the emissions from deep‐level defects in h‐BN are SPEs and can be characterized directly in the spectrum as a zero‐phonon line (ZPL), which often possesses an impressively narrow bandwidth.

The SPEs from h‐BN located near 2 eV can be categorized into two groups, depending on three spectral characteristics (Figure 3f).^[^ ^126^ ^]^ First, group 1 features ZPL energies in the range from 1.82 to 2.16 eV, corresponding to the wavelength range from 575 to 681 nm; group 2 features ZPL energies in the range from 1.63 to 1.82 eV, corresponding to the wavelength range from 681 to 762 nm. Second, the ZPLs in group 1 are relatively broader with asymmetric broadening toward lower energy, while those in group 2 have narrower and more symmetric bands. Third, both of the groups provide a phonon sideband (PSB) located at a lower energy of 160 ± 5 meV from their ZPL energy, but the intensities of the PSBs in group 1 are significant and those in group 2 are relatively small. Because these two groups of SPEs exhibit different spectral characteristics, they presumably result from different types of defects; several attempts have been made to theoretically determine the origin of these SPEs according to their emission peak positions and spectral line shapes. Calculations have suggested that the group‐1 SPEs in h‐BN might possibly be related to intrinsic defects [e.g., V_N_ (a N atom vacancy)^[^ ^125^ ^]^ or N_B_V_N_ (a N atom occupying a B site and a vacancy existing at the N site)],^[^ ^125^ ^]^ while the group‐2 SPEs might correspond to C_B_V_N_ defects (a substitutional C atom impurity at the B site and a N vacancy);^[^ ^134^ ^]^ B_i_, C_i_, and O_i_ interstitials;^[^ ^135^ ^]^ or several hydrogen or oxygen complexes.^[^ ^135^ ^]^ Nevertheless, discrepancies exist between the theoretical and experimental results, making it a challenge to identify the precise nature of these defects when investigating the SPEs of h‐BN samples. Such variances result from the fact that the SPEs are involved with localized atomic systems (i.e., atomic defects) that are very sensitive to differences in the dielectric environment,^[^ ^126^ ^]^ charge dynamics,^[^ ^129^ ^]^ local strain,^[^ ^130^ ^]^ and temperature around the defects.^[^ ^128^ ^]^ Therefore, applying SPEs to identify the nature of the defects in h‐BN samples will require further investigations of their properties (e.g., emission wavelengths, polarization of emitted light, and absorption behavior) and their correlation to the nature of the defects produced under various growth conditions.

## 6 Probing Strain in 2D Materials

Strain is readily introduced into 2D materials during the fabrication of 2D material‐based devices using, for example, mechanical exfoliation, epitaxial growth, transfer, and integration to non‐flat substrates. The presence of strain in a 2D material means that there are changes in the bond lengths, angles, and strengths, and relative positions, of some of the atoms; therefore, the interactions between the electrons and lattice atoms in the 2D material are varied, leading to significant effects on their physical properties.^[^ ^136^ ^]^ For example, a bandgap opening of approximately 300 meV was found for monolayer graphene under 1% uniaxial tensile strain.^[^ ^137^ ^]^ The electronic band structures of several 2D materials can be altered in the presence of strain; for example, the bandgap energies of TMDCs and InSe decrease, while those of BP increase, under uniaxial or biaxial tensile strain.^[^ ^138^ ^]^ Some semiconducting 2D materials undergo a direct‐to‐indirect or indirect‐to‐direct transition when applying strain, strongly influencing their optical properties. The transport of electrons and thermal phonons along the direction of applied strain in 2D materials can also be affected, leading to changes in electrical or thermal conductivity.^[^ ^136^ ^]^ Accordingly, the presence of strain in 2D materials will strongly influence their properties and, thereby, affect the performance of corresponding 2D material‐based devices. Therefore, for process optimization, there is a need to probe the magnitudes and spatial directions of these strains after device fabrication.

Optical inspection techniques can be applied to probe the built‐in strain in 2D materials, as long as the relationship has been established between the strain and the optical signatures. To investigate such relationship, it is necessary to apply controllable strain to 2D materials. Several methods have been proposed to readily introduce strain to 2D materials.^[^ ^136^ ^]^ Among them, deforming a polymer substrate upon which 2D materials are attached is the most commonly used. Mechanically pulling, squeezing, or bending from the two sides of the substrate can transfer the uniaxial tensile or compressive strain applied to the substrate onto the attached 2D material, if there is no slippage and decoupling between the polymer and the 2D material. Thus, the strain‐induced optical signatures from 2D materials can then be investigated systematically.

**Figure 4:**
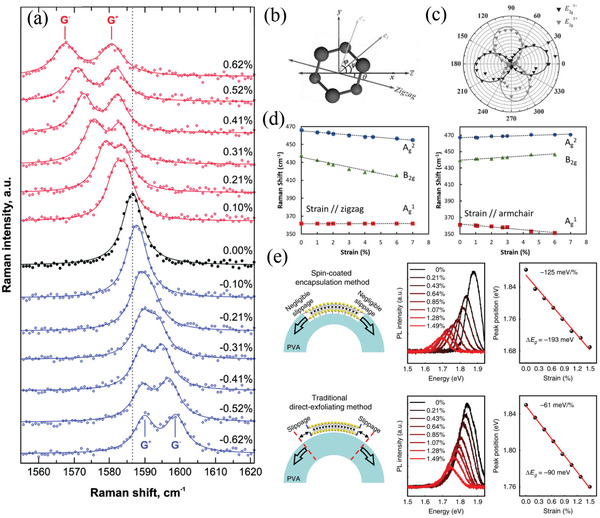
Strain‐dependent optical properties of 2D materials. a) Raman spectra of graphene (G band) recorded under different degrees of uniaxial strain (positive: tensile strain; negative: compressive strain); solid curves are the best Lorentzian fittings of the experimental data. Reproduced with permission.^[^ ^140^ ^]^ Copyright 2010, American Chemical Society. b) Schematic representation of the configuration in polarization‐dependent Raman spectroscopy; $\overset{⇀}{\varepsilon }$ is the direction of applied stain along the *x*‐axis; $\overset{⇀}{e_{i}}$ and $\overset{⇀}{e_{s}}$ are the polarizations of incident and scattered light, respectively. c) Polarization‐dependent intensities of the $E_{2g}^{1+}$ and $E_{2g}^{1-}$ modes from monolayer MoS_2_ plotted with respect to *φ*.; the polarization of incident light is fixed to be aligned along the direction of applied strain, so that *ψ* is zero. b,c) Reproduced with permission.^[^ ^142^ ^]^ Copyright 2013, Wiley‐VCH. d) Raman modes of BP under uniaxial tensile strain along zigzag (left) and armchair (right) directions; dashed lines are linear fitted results. Reproduced with permission.^[^ ^149^ ^]^ Copyright 2018, American Chemical Society. e) PL spectra (middle) of monolayer MoS_2_, prepared using different methods, under uniaxial tensile strain. Left: Schematic representations; right: PL peak positions plotted with respect to strain (red lines: linear fitted results). Reproduced under the terms and conditions of the CC‐BY license.^[^ ^150^ ^]^ Copyright 2020, The Authors. Published by Nature Publishing Group.

Typically, the presence of tensile (compressive) strain decreases (increases) the bond length and then softens (stiffens) the lattice vibrations; Raman spectra, therefore, can be used to reveal the optical signature when probing strain in 2D materials. The Raman spectra of uniaxially strained graphene,^[^ ^139^, ^140^, ^141^ ^]^ TMDCs,^[^ ^142^, ^143^, ^144^ ^]^ h‐BN,^[^ ^145^ ^]^ InSe,^[^ ^146^ ^]^ and BP^[^ ^147^, ^148^, ^149^ ^]^ on polymer substrates have been investigated experimentally and theoretically; some of their Raman spectral peak positions have been found to shift linearly with respect to the applied strain, such that the peak shift can be used to identify the magnitude of the strain in the 2D material. Interestingly, when uniaxial strain is applied to graphene, TMDCs, and h‐BN, their in‐plane E_2g_ vibration Raman modes (e.g., the G band of graphene;^[^ ^139^, ^140^ ^]^ the $E_{2g}^{1}$ modes of MoS_2_,^[^ ^143^ ^]^ WS_2_,^[^ ^144^ ^]^ and WSe_2_;^[^ ^144^ ^]^ the G band of h‐BN)^[^ ^145^ ^]^ split into two singlet sub‐modes, due to breaking of lattice symmetry under the uniaxial strain. These sub‐modes are denoted as G^+^ and G^‐^ or $E_{2g}^{1+}$ and $E_{2g}^{1-}$, according to their energies (**Figure** **4**a displays an example of the splitting of the graphene Raman spectral G band under uniaxial strain).^[^ ^140^ ^]^ The G^+^ and $E_{2g}^{1+}$ modes correspond to lattice vibrations along the direction perpendicular to the applied strain; as a result, they experience smaller softening (downshifts) or stiffening (upshifts) when being strained, such that they exhibit smaller shift rates. In contrast, the G^‐^ and $E_{2g}^{1-}$ modes are lattice vibration modes along the direction parallel to the applied strain; therefore, they demonstrate shift rates greater than those of the G^+^ and $E_{2g}^{1+}$ modes. Accordingly, the shifts of the G^‐^ and $E_{2g}^{1-}$ modes can be chosen as a more sensitive indicator for probing the magnitude of uniaxial strain in graphene, TMDCs, and h‐BN. The shift rates under tensile strain have been reported to be approximately ‐30 cm^‐1^/% for the graphene G^‐^ band;^[^ ^140^ ^]^ to extend from ‐1 to ‐3 cm^‐1^/% or ‐7 cm^‐1^/% for the $E_{2g}^{1-}$ modes of MoS_2_, WS_2_, and WSe_2_;^[^ ^143^, ^144^, ^150^ ^]^ to be approximately ‐25 cm^‐1^/% for the G^‐^band of h‐BN;^[^ ^145^ ^]^ and to be approximately ‐3 cm^‐1^/% for the $E_{2g}^{1}$, $A_{1g}^{1}$(LO), and $A_{1g}^{2}\;$modes of InSe.^[^ ^146^ ^]^ Notably, the effect of uniaxial strain on the Raman spectral peak shifts for BP is more complex, and will be discussed later.

The splitting of the in‐plane E_2g_ vibration mode results from the presence of uniaxial strain that breaks the lattice symmetry; therefore, these submodes should be polarization‐dependent and related to the direction of the uniaxial strain with respect to the crystalline orientation. Accordingly, polarization‐dependent Raman spectroscopy could possibly provide information about the spatial direction of the built‐in strain in 2D materials. The intensities of the G^+^ ($E_{2g}^{1+}$) and G^‐^ ($E_{2g}^{1-}$) modes of graphene (TMDCs) in polarization‐dependent Raman spectra can be expressed using the following equations (see Figure 4b for the configuration):^[^ ^139^, ^142^ ^]^

(4) $$I\left(G^{+}\;\mathrm{or}\;E_{2g}^{1+}\right)\;\propto \;d^{2}\cos^{2}\left(\phi \;+\;\psi \;+\;3\theta \right)$$

(5) $$I\left(G^{-}\;\;\mathrm{or}\;E_{2g}^{1-}\right)\;\propto \;d^{2}\sin^{2}\left(\phi \;+\;\psi \;+\;3\theta \right)$$

where *d* is the Raman tensor element of the Raman vibration mode; *φ* and *ψ* are the polarization angles of the incident and scattered light, respectively, with respect to the direction of applied strain; and *θ* is the angle between the direction of applied strain and the zigzag direction of the crystal lattice. By measuring the polarization‐dependent intensities of both the G^+^ ($E_{2g}^{1+}$) and G^‐^ ($E_{2g}^{1-}$) modes with respect to the polarization angles of the incident and scattered light, the angle of *θ* can be obtained—that is, the crystalline orientation can be identified (Figure 4c).^[^ ^142^ ^]^ In other words, if the crystalline orientation is pre‐determined, the spatial direction of the strain in graphene or TMDCs can be characterized using polarization‐dependent Raman spectroscopy.

Uniaxial strain in BP results in Raman spectral characteristics distinct from those of other 2D materials, due to its anisotropic structure. Instead of splitting of the vibration mode, the three main characteristic Raman spectral modes of BP shift oppositely, with uniaxial strain along the zigzag and armchair directions (Figure 4d).^[^ ^149^ ^]^ The B_2g_ and $A_{g}^{2}$ modes downshift linearly with significant shift rates (‐4.7 and ‐1.9 cm^‐1^/%, respectively) for uniaxial tensile strain in the zigzag direction, while the $A_{g}^{1}$ mode exhibits a negligible shift. On the other hand, when a uniaxial tensile strain is present along the armchair direction, the $A_{g}^{1}$ mode downshifts (shift rate is ‐2.0 cm^‐1^/%), while the B_2g_ and $A_{g}^{2}$ modes undergo upshifts (shift rates of 2.4 and 1.6 cm^‐1^/%, respectively). Accordingly, the strain‐induced peak shifts of the Raman spectral modes of BP are strongly dependent not only on the magnitude but also on the direction of the strain; therefore, the peak shifts of the three Raman spectral modes can be regarded as a direct signature of the built‐in strain in an arbitrary direction in BP samples.^[^ ^147^ ^]^

So far, we have reviewed the strain‐induced Raman spectral responses of several 2D materials and discussed how they can be used to probe the magnitude and direction of the built‐in uniaxial strain. The effects of biaxial strain on the Raman spectral signatures of 2D materials should also be taken into consideration because the biaxial strain is usually introduced during thermal growth processes (e.g., CVD) as a result of differences in the thermal expansion coefficients between the 2D materials and their supporting substrates. Three common methods have been used to apply biaxial strain to 2D materials.^[^ ^136^ ^]^ First, 2D materials can be attached to a piezoelectric material, such that controllable strain can be introduced mechanically by applying an electric field. Second, 2D materials can be strained biaxially by using a substrate that can readily be expanded thermally; the thermal effect on the Raman spectral shifts should be measured on a substrate that undergoes almost no thermal expansion and then subtracted to get the pure biaxial strain contribution. Third, 2D materials can be transferred onto pre‐patterned substrates featuring trenches or holes, thereby functioning as a deformable suspended membrane; the biaxial strain can be introduced by applying different gas pressures in the cavities under the suspended 2D materials. Using these methods, the biaxial strain responses of graphene, TMDCs, and h‐BN have been studied, providing shift rates under biaxial tensile strain of approximately ‐60 cm^‐1^/% for the D and G bands and approximately ‐160 cm^‐1^/% for the 2D band of graphene;^[^ ^151^ ^]^ from ‐0.1 to ‐1.5 cm^‐1^/% for the A_1g_ mode and from ‐2.7 to ‐7.2 cm^‐1^/% for the $E_{2g}^{1}$ mode of TMDCs;^[^ ^152^ ^]^ and of approximately ‐39 cm^‐1^/% for the G band of h‐BN.^[^ ^145^ ^]^

Because strain alters the electronic band structures of 2D materials, their bandgap energies will change upon the application of strain, such that their related optical properties can be used as signatures for probing the built‐in strain. The effects of uniaxial and biaxial strain on the bandgap energies of TMDCs^[^ ^150^, ^153^, ^154^, ^155^ ^]^ and InSe^[^ ^138^, ^146^, ^156^ ^]^ have been studied using PL spectroscopy, and those of BP^[^ ^157^, ^158^ ^]^ have been investigated using IR extinction spectroscopy. The changes in bandgap energy for group‐IV monochalcogenides have, however, been investigated only theoretically.^[^ ^159^ ^]^ When uniaxial strain is applied to TMDCs, InSe, and BP, their bandgap energies shift linearly upon increasing the strain. Surprisingly, the shift rates are almost the same along the zigzag and armchair directions—even for BP, which exhibits intrinsic anisotropy (although the shifts of the bandgap energies for group‐IV monochalcogenides exhibit anisotropic characteristics, we exclude their discussion because they have yet to be investigated experimentally).^[^ ^156^, ^157^, ^160^ ^]^ The orientation‐independent behavior of BP can be attributed to the strain affecting the bandgap energies not only along its in‐plane zigzag and armchair directions but also along its out‐of‐plane direction, as expressed by the following equation:^[^ ^157^ ^]^

(6) $$\Delta E_{g}=4.1\varepsilon_{\mathrm{AC}}+5.7\varepsilon_{\mathrm{ZZ}}-12.9\varepsilon_{\mathrm{OP}}$$

where ΔE_g_ is the change in bandgap energy and *ε*_AC_, *ε*_ZZ_, and *ε*_OP_ are the strains along the armchair, zigzag, and out‐of‐plane directions, respectively. When the tensile strain is present along the armchair direction, the out‐of‐plane direction will be compressed, resulting in an additional increase in the bandgap energy because the coefficient of *ε*_OP_ is negative. In contrast, the tensile strain along the zigzag direction will lead to expansion along the out‐of‐plane direction, due to its special puckered structure, thereby decreasing the bandgap energy. Accordingly, the different effects of strain along the armchair and zigzag directions will be canceled out, due to opposite Poisson effects occurring along these directions. Because of such orientation independence, only the magnitude of the built‐in strain can be probed; the spatial direction is difficult to determine using PL spectroscopy. To briefly summarize, the shift rates of the bandgap energies with uniaxial strain are approximately in the range from ‐30 to ‐120 meV per % for TMDCs,^[^ ^150^, ^153^ ^]^ approximately in the range from ‐100 to ‐150 meV per % for InSe,^[^ ^146^, ^156^ ^]^ and approximately 120 meV per % for BP (note that the bandgap energy is blue‐shifted for BP, but red‐shifted for the other 2D materials).^[^ ^157^ ^]^ The effect of biaxial strain on the bandgap energies is a combination of the contributions of uniaxial strain along both the zigzag and armchair directions; thus, the shifts in the bandgap energies for biaxial strain are approximately twice those for uniaxial strain. The shift rates with biaxial strain have been reported to be approximately in the range from ‐90 to ‐120 meV per % for TMDCs,^[^ ^154^, ^155^ ^]^ approximately ‐200 meV per % for InSe,^[^ ^138^ ^]^ and approximately 220 meV per % for BP.^[^ ^158^ ^]^

The changes in the electronic band structures of 2D materials also influence the magnitudes of their direct and indirect bandgaps; thus, their affected PL peak intensities can also be used as indicators for probing the strain. For example, when tensile strain is introduced to monolayer MoS_2_,^[^ ^161^ ^]^ monolayer WS_2_,^[^ ^162^ ^]^ and few‐layer InSe,^[^ ^146^ ^]^ all of which are originally direct bandgap materials, the decrease in their indirect bandgap with strain is faster than that of their direct bandgap; as a result, the spectral weight of the direct transition gradually decreases upon increasing the strain, such that their main characteristic PL spectral peaks (corresponding to the direct transition) will decrease in intensity. Further increasing the strain can cause the energy of the indirect bandgap to become smaller than that of the direct bandgap,^[^ ^161^, ^162^ ^]^ leading to a direct‐to‐indirect transition and, possibly, the appearance of a PL spectral peak corresponding to an indirect transition. On the other hand, some indirect bandgap materials (e.g., bilayer WSe_2_^[^ ^163^ ^]^ and bilayer MoTe_2_)^[^ ^164^ ^]^ will undergo an indirect‐to‐direct transition when tensile strain is present. The intensity of the PL spectral peak that corresponds to the direct bandgap will increase upon increasing the strain, such that this relationship can be used also to identify the magnitude of strain.

When studying the relationship between the applied strain and the optical signatures of 2D materials, two factors should be taken into consideration. First, to apply strain to 2D materials, the most common strategy has been to transfer them onto the surface of a polymer substrate that can be bent or stretched mechanically. By deforming such a polymer substrate, the strain would be expected to transfer to the attached 2D materials. Nevertheless, because vdW forces between the 2D materials and the substrate are weak, there usually exists unavoidable slippage during the deformation, such that the strain applied to the substrate cannot be transferred effectively to the attached 2D materials. More specifically, the actual strain applied to the 2D materials will be lower than expected, thereby leading to underestimations of the shift rates of the optical signatures. To address this issue, a spin‐coated encapsulation method has, for example, been proposed to strengthen the interaction between the 2D material (e.g., monolayer MoS_2_) and the substrate, thereby ensuring there no slippage occurs and almost 100% of the strain can be transferred. As displayed in Figure 4e,^[^ ^150^ ^]^ the actual strain‐induced optical signatures (PL spectral peak shifts) obtained through spin‐coated encapsulation can be compared with those obtained from a system exhibiting slippage. That study highlights the fact that slippage between the 2D materials and the substrate during deformation should be avoided when investigating the effects of strain in 2D materials.

Second, when investigating the relationship between uniaxial strain and optical signatures, the Poisson effect of the substrate will influence the strain‐induced optical signatures from the 2D materials, such that the true relationship cannot be determined. The Poisson effect occurs when uniaxial strain is applied to materials (here, the deformable substrate) and compression or expansion is induced in the direction perpendicular to the direction of the applied strain, with the induced deformation expressed as the Poisson ratio. As a result, transverse strain from the Poisson effect will give rise to an additional contribution to the strain‐induced optical signatures;^[^ ^154^ ^]^ therefore, a polymer substrate having a small Poisson ratio should be used to obtain the actual optical signatures with respect to the applied uniaxial strain. Briefly, slippage between the 2D material and the substrate and the Poisson effect of the substrate should both be factored when optically inspecting the effects of strain in 2D materials.

## 7 Monitoring the Type and Concentration of Doped Carriers in 2D Materials

Doping is a key process in modern semiconductor technologies. Through n‐ and p‐type doping of semiconducting materials (e.g., silicon and III‐V semiconductors), p–n junctions and transistors can be fabricated as important components of several electronic and optoelectronic devices. Regarding the fabrication of 2D material–based devices, doping also plays an important role for modulating the electrical, optical, and structural properties of the 2D materials; due to their atomically thin nature, however, traditional dopant diffusion and implantation are not suitable approaches. Several methods have been proposed for doping 2D materials, including electrostatic,^[^ ^165^ ^]^ contact,^[^ ^166^ ^]^ chemical,^[^ ^167^ ^]^ and substitutional^[^ ^168^, ^169^ ^]^ doping. Using these methods, the doping of 2D materials can be achieved by controlling their carrier concentration through electrostatic effects, charge transfer, changes in the electronic band structure, or replacement of the lattice atoms with atoms having a different number of electrons. Because the electrical and optical properties of 2D materials—and, hence, the performance of their resultant devices—are determined by the types and concentrations of their doped carriers, non‐destructive techniques for their characterization after doping have received much attention.

**Figure 5:**
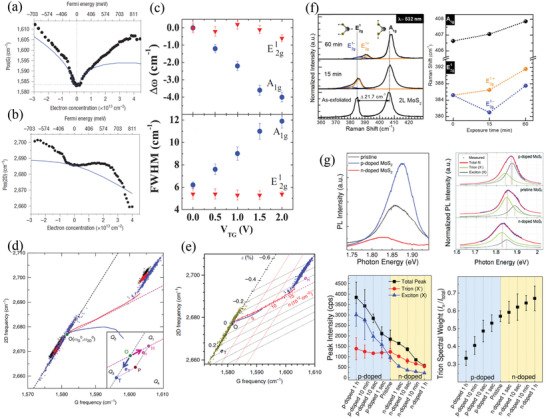
Doping‐related optical properties of 2D materials. Peak positions of a) graphene Raman G and b) 2D bands, plotted with respect to the degrees of electron and hole doping; solid curves: simulated results. a,b) Reproduced with permission.^[^ ^165^ ^]^ Copyright 2008, Nature Publishing Group. c) Peak shifts (top) and full widths at half maximum (FWHMs; bottom) of the A_1g_ and $E_{2g}^{1}$ modes of monolayer MoS_2_ plotted with respect to the applied gate‐voltage (*V*_TG_). Reproduced with permission.^[^ ^172^ ^]^ Copyright 2012, American Physical Society. d) Correlation analysis of the two Raman spectral bands of the mechanically exfoliated graphene samples, to distinguish the strain and doping effects via the vector model (see inset); data were obtained from three samples before (+) and after (×) thermal annealing at 400 °C. e) Correlation analysis of (*ω*_G_, *ω*_2D_) of a graphene sample before and after thermal annealing. d,e) Reproduced with permission.^[^ ^181^ ^]^ Copyright 2012, Nature Publishing Group. f) Measured Raman spectra (left) and peak positions (right) of bilayer MoS_2_ prepared with different N_2_ plasma exposure times. Reproduced with permission.^[^ ^178^ ^]^ Copyright 2016, American Chemical Society. g) Measured PL spectra (upper left), peak deconvolution analysis (upper right and lower left), and the trion spectral weight (lower right) of monolayer MoS_2_ before and after n‐ and p‐doping. Reproduced with permission.^[^ ^177^ ^]^Copyright 2018, Wiley‐VCH.

In doped 2D materials, the electron–phonon interaction and the shift in the Fermi level induced by excess charge carriers give rise to changes in their Raman and PL spectral characteristics; therefore, Raman and PL spectroscopies can be useful tools for monitoring the types and concentrations of the doped charges. Here, we first discuss the use of Raman spectroscopy for characterizing doped 2D materials. The relationships between the charge carriers and the Raman spectra of graphene,^[^ ^165^, ^170^, ^171^ ^]^ MoS_2_,^[^ ^172^ ^]^ WS_2_,^[^ ^173^ ^]^ and MoSe_2_^[^ ^174^ ^]^ have all been investigated after electrostatic doping. The electrostatic doping of these 2D materials has been performed by inducing charge carriers capacitively coupled to the back‐gate (electrically back‐gating)^[^ ^171^, ^173^, ^174^ ^]^ or to the statistical space charge accumulated around the 2D material–electrolyte interface (electrochemically top‐gating).^[^ ^165^, ^170^, ^172^ ^]^ This doping method has several attractive features: it is non‐destructive, stable, reversible, and durable. Most importantly, the carrier concentration can be evaluated directly by its relationship to capacitance coupling, allowing the recording of pure charge carrier‐dependent Raman spectra of doped 2D materials with respect to the charge concentration. After applying this doping technique, several important features can appear in the Raman spectra of doped graphene. First, the Raman G band of doped graphene undergoes an upshift upon increasing the carrier concentration (by varying the gate voltage) for both electron and hole doping (**Figure** **5**a)^[^ ^165^ ^]^ The FWHM of the G band also decreases for both electron and hole doping, and then rapidly saturates when the Fermi level shift induced by doping is larger than half of the phonon energy. Therefore, the G band alone is not a suitable indicator for determining the doping of graphene. Second, compared with the Raman G band, the graphene 2D band exhibits a different dependence on doping (Figure 5b).^[^ ^165^ ^]^ When graphene is electron‐doped, the peak position of the 2D band does not shift significantly (<1 cm^‐1^) until the electron concentration is greater than 2.5 × 10^13^ cm^‐2^ and then it downshifts upon further increasing the electron concentration; for hole doping, the 2D band gradually upshifts upon increasing the hole concentration. Furthermore, the intensity ratio of the 2D and G bands decreases for both electron and hole doping.^[^ ^165^, ^171^ ^]^ Briefly, considering both the directions and degrees of peak shifting of the graphene G and 2D bands can be used to identify its type of doping (n‐ or p‐type) and the carrier concentration. In addition, the carrier concentration in graphene can also be determined from the intensity ratio of the 2D and G bands.

In the case of electrostatic doping of MoS_2_,^[^ ^172^ ^]^ WS_2_,^[^ ^173^ ^]^ and MoSe_2_,^[^ ^174^ ^]^ it has been found experimentally and theoretically that their out‐of‐plane A_1g_ vibration modes downshift (upshift) and broaden (sharpen) upon electron (hole) doping (Figure 5c), while their in‐plane $E_{2g}^{1}$ vibration modes are insensitive to charge carriers.^[^ ^172^, ^173^, ^174^, ^175^ ^]^ This charge‐dependent behavior for TMDCs can be used to monitor their type and carrier concentration of doping, and is quite different from the effect of strain on Raman spectra of TMDCs, in which the $E_{2g}^{1}$ mode varies while the A_1g_ mode is insensitive to strain,^[^ ^143^, ^144^ ^]^ making it possible to distinguish the contributions of strain and doping on the Raman spectra of TMDCs. The effects of strain and doping on the Raman spectra of TMDCs are discussed below.

Having established the relationship between carrier concentration and changes in the Raman spectra of graphene and TMDCs, the effects of contact doping,^[^ ^176^ ^]^ chemical doping,^[^ ^177^ ^]^ and substitutional doping^[^ ^168^, ^169^, ^178^ ^]^ on graphene and TMDCs have been studied by investigating their Raman spectra. Notably, the chemical doping and substitutional doping of InSe^[^ ^179^ ^]^ and BP^[^ ^167^, ^180^ ^]^ have also been characterized using Raman spectroscopy, even though their pure charge carrier–dependence has yet to be established. The Raman spectral modes of InSe and BP downshifted (upshifted) in the same direction as the shift in the A_1g_ mode of TMDCs with electron (hole) doping, which tends to soften (stiffen) the vibrations. Nevertheless, rigorous quantification of the doping carrier concentrations will still rely in future on further establishing their pure charge carrier–dependent Raman spectra.

In Raman spectral characterizations of the effects on contact doping, chemical doping, and substitutional doping of graphene and TMDCs, the doping processes are usually accompanied, unexpectedly, by the introduction of strain, especially for substitutional doping, which replaces the original lattice atoms with atoms of different sizes, such that lattice distortion will occur. Therefore, it is necessary to distinguish between the effects of strain and charge carriers on the Raman spectral signatures. For graphene, it has been reported that the peak positions of the G and 2D bands are strongly related to uniaxial or biaxial strain.^[^ ^139^, ^140^, ^141^, ^151^ ^]^ Furthermore, these peak positions depend significantly on the carrier concentration, as above discussed.^[^ ^165^ ^]^ According to these studies, pure strain or charge carrier effects in graphene can be characterized by the peak positions of the G and 2D bands; when both strain and excess charge carriers are introduced into graphene, however, such bimodal dependence makes it more complicated to determine the respective effects on the Raman spectral signatures. To separate the effects of strain and charge carriers, a vector model has been proposed based on a correlation analysis of the two Raman spectral bands of mechanically exfoliated graphene samples (Figure 5d).^[^ ^181^ ^]^ The model was begun by measuring the Raman spectra of suspended freestanding graphene, and the peak positions of its main signals (*ω* ^0^_G_ and *ω* ^0^_2D_ for the G and 2D bands, respectively, of freestanding graphene) were set to be of strain‐ and charge carrier‐free origin, as denoted by the green circle. When a randomly oriented uniaxial strain was present in graphene, both the peak positions of the G and 2D bands (*ω*_G_ and *ω*_2D_, respectively) downshifted (upshifted) linearly upon increasing the tensile (compressive) strain, and ratio of the shift rates, denoted by (Δ*ω*_2D_/Δ*ω*_G_)*ε*, was expected to be 2.2 ± 0.2 (black dashed line in Figure 5d), in agreement with the values obtained from previous studies. Regarding the effect of the charge carriers, the dependence on *ω*_G_ and *ω*_2D_ for graphene doped with varying concentration of holes and electrons is represented by the red and blue lines, respectively. This dependence was obtained from a study of the relationship between the Raman spectra and the charge carriers doped through electrostatic doping,^[^ ^171^ ^]^ similar to the results discussed above (Figure 5a,b).^[^ ^165^ ^]^ Because many previous studies have demonstrated that hole doping is dominant for pristine and annealed graphene, if considering only the hole doping, the *ω*_G_ and *ω*_2D_ will follow quasi‐linearity with a ratio of the shift rates, denoted as (Δ*ω*_2D_/Δ*ω*_G_)_hole_, which is expected to be 0.75 ± 0.04 (magenta dashed line in Figure 5d). Accordingly, once the quantities of the shifts in *ω*_G_ and *ω*_2D_ for both strain and charge carriers are determined, the “strain‐free” unit vector ***e*_H_** (for hole doping) and “charge‐neutral” unit vector ***e*_T_** (for tensile strain) can be established (see inset to Figure 5d). Therefore, for any arbitrary *ω*_G_ and *ω*_2D_ (denoted as vector of **OP**), the vector can be decomposed into **OH** and **OT**, representing the effects of the charge carrier and the strain, respectively. Figure 5e displays an example of the separation of the strain and charge carrier effects by using the vector model.^[^ ^181^ ^]^ The strain of the graphene sample prior to annealing ranged from ‐0.2 to 0.4% and the hole concentration was less than 1.0 × 10^12^ cm^‐2^ (in khaki), while the strain ranged from ‐0.3 to 0% and the hole concentration was 1.4 ± 0.1 × 10^13^ cm^‐2^ after annealing at 400 °C (in blue).

In previous studies, the $E_{2g}^{1}$ mode of TMDCs was found to be very sensitive to strain, while the A_1g_ mode was insensitive;^[^ ^143^, ^144^, ^150^, ^152^ ^]^ on the other hand, the A_1g_ mode was influenced by excess charge carriers, while the $E_{2g}^{1}$ mode was not affected.^[^ ^172^, ^173^, ^174^, ^175^, ^182^ ^]^ According to these relationships, distinguishing the effects of strain and charge carriers on the Raman spectra of TMDCs will be much easier than in the case of graphene, because each of the $E_{2g}^{1}$ and A_1g_ modes can be regarded directly as the indicator for strain and charge carriers, respectively. Figure 5f presents an example of the substitutional nitrogen (N) doping of MoS_2_ studied using Raman spectroscopy.^[^ ^178^ ^]^ The A_1g_ mode of the N‐doped MoS_2_ underwent an upshift and the FWHM sharpened upon increasing the exposure time to the N_2_ plasma, representative of the p‐type doping of MoS_2_. This result is consistent with the number of electrons in nitrogen being less than that in sulfur, suggesting a p‐type doping of MoS_2_. On the other hand, when the sulfur atoms were substituted by nitrogen atoms, the $E_{2g}^{1}$ mode split into two singlet subbands, denoted as $E_{2g}^{1+}$ and $E_{2g}^{1-}$, and both of them became upshifted upon increasing the nitrogen atom concentration. Such changes in Raman spectra reveal the presence of compressive uniaxial strain, and also agree with the fact that the atomic radius of nitrogen is smaller than that of sulfur; hence, the formation of Mo–N bonds could possibly generate compressive strain. Therefore, Raman spectroscopy is indeed a useful tool for characterizing the strain and excess charge carriers in TMDCs.

Next, we discuss the use of PL spectroscopy for characterizing the doping in 2D materials. As mentioned above, the PL spectra of semiconducting 2D materials (e.g., TMDCs) feature three dominant peaks for the neutral free exciton emission (X_0_), the negatively charged exciton or trion emission (X^‐^), and the defect‐bound exciton emission (X_B_). The defect‐bound exciton emission arises from the localization of neutral excitons bound by defects; thus, the intensity of the X_B_ peak can be related to the defect density. On the other hand, the negatively charged exciton (or trion emission) results from the binding of neutral excitons to excess electrons, which originate from the n‐type doping. The relationship between the intensities of the X_0_ and X^‐^ peaks can, therefore, provide direct information about the doping carrier concentration in a 2D material.^[^ ^183^, ^184^ ^]^ Let us consider MoS_2_ as an example; pristine MoS_2_ is an intrinsic n‐type doping semiconductor, due to the presence of native sulfur vacancies; thus, its PL spectra can be decomposed into the exciton peak (X_0_) and trion peak (X^‐^), fitted by Lorentzian peaks (Figure 5g).^[^ ^177^ ^]^ When the MoS_2_ is p‐doped, the PL peak increases in intensity and the maxima are slightly shifted to higher energy. This phenomenon is due to the fact that hole doping can neutralize the negative carriers, such that fewer trions are formed in MoS_2_. As a result, the PL spectra of p‐type doped MoS_2_ become increasingly dominated by the exciton emission (X_0_), which possess higher radiative quantum efficiency and higher emission energy, and the spectral weight of the trion emission ($I_{X^{-}}$/*I*_total_) decreases upon increasing the concentration of p‐type doping (Figure 5g). In contrast, n‐type doped MoS_2_ will cause $I_{X^{-}}$/*I*_total_ to increase upon increasing the concentration of n‐type doping; therefore, the intensity of the signals in its PL spectra decrease and the peak maxima shift to lower energy. In other words, the PL spectral intensity is enhanced after p‐type doping and quenched after n‐type doping, and the doping concentration can be related to the PL spectral weight of the trion peak (X^‐^). More specifically, the electron concentration in MoS_2_ can be evaluated from the PL spectral weight of the trion peak ($I_{X^{-}}/I_{\mathrm{total}}$) using the mass action law:^[^ ^183^ ^]^

(7) $$\frac{I_{X^{-}}}{I_{\mathrm{total}}}\;=\;\frac{\frac{\gamma_{\mathrm{tr}}}{\gamma_{\mathrm{ex}}}\frac{N_{X^{-}}}{N_{X}}}{1+\frac{\gamma_{\mathrm{tr}}}{\gamma_{\mathrm{ex}}}\frac{N_{X^{-}}}{N_{X}}}\;\approx \;\frac{4\;\times \;10^{-14}n_{\mathrm{el}}}{1\;+\;4\;\times \;10^{-14}n_{\mathrm{el}}}$$

where *γ*_ex_ and *γ*_tr_ are the radiative decay rates of the exciton and trion, respectively; N_X_ and $N_{X^{-}}$ are the populations of the exciton and trion, respectively; and *n*_el_ is the concentration of doped electrons. Finally, it is notable that the same trend in the PL spectra related to p‐ or n‐type doping can also be observed for other TMDCs, including WS_2_, MoSe_2_, and WSe_2_.^[^ ^177^ ^]^

## 8 Studying Carrier Dynamics in 2D Materials

When applying 2D materials for photovoltaics, photodetectors, and light‐emitting devices, ultrafast carrier dynamics (involving carrier–photon, carrier–carrier, and carrier–phonon interactions) will play a key role in determining the performance of such 2D material‐based devices. Understanding the carrier dynamics will be helpful when manipulating the carrier behavior (e.g., the separation and relaxation of the photo‐excited electron/hole pairs) to ensure rapid response times or high quantum efficiencies. As a result, investigating the carrier dynamics of 2D materials is necessary to improve the performance of those optoelectronic devices. To measure the carrier dynamics, two types of ultrafast spectroscopy have been applied: time‐resolved photoluminescence (TRPL) spectroscopy^[^ ^185^ ^]^ and pump‐probe spectroscopy.^[^ ^186^ ^]^ TRPL records spectroscopy the emissions from samples with respect to time after the pulse laser excitation; thus, it is a method that can provide the emission lifetime directly during the radiative recombination of the photo‐excited carriers. On the other hand, in pump‐probe spectroscopy, the samples are first excited by a pump pulse to generate photo‐excited carriers. The presence of the photoexcited carriers will induce changes in the optical constants (e.g., refractive indices and extinction coefficients), leading to changes in the reflectance, transmittance, and absorptance; these changes with respect to time and even wavelength are measured by a delayed probe pulse, which is generated from the same pulse laser as the pump pulse. Accordingly, even the optically inactive or dark states involved in the relaxation of the photoexcited carriers can be monitored by pump‐probe spectroscopy, but not by TRPL spectroscopy.

**Figure 6:**
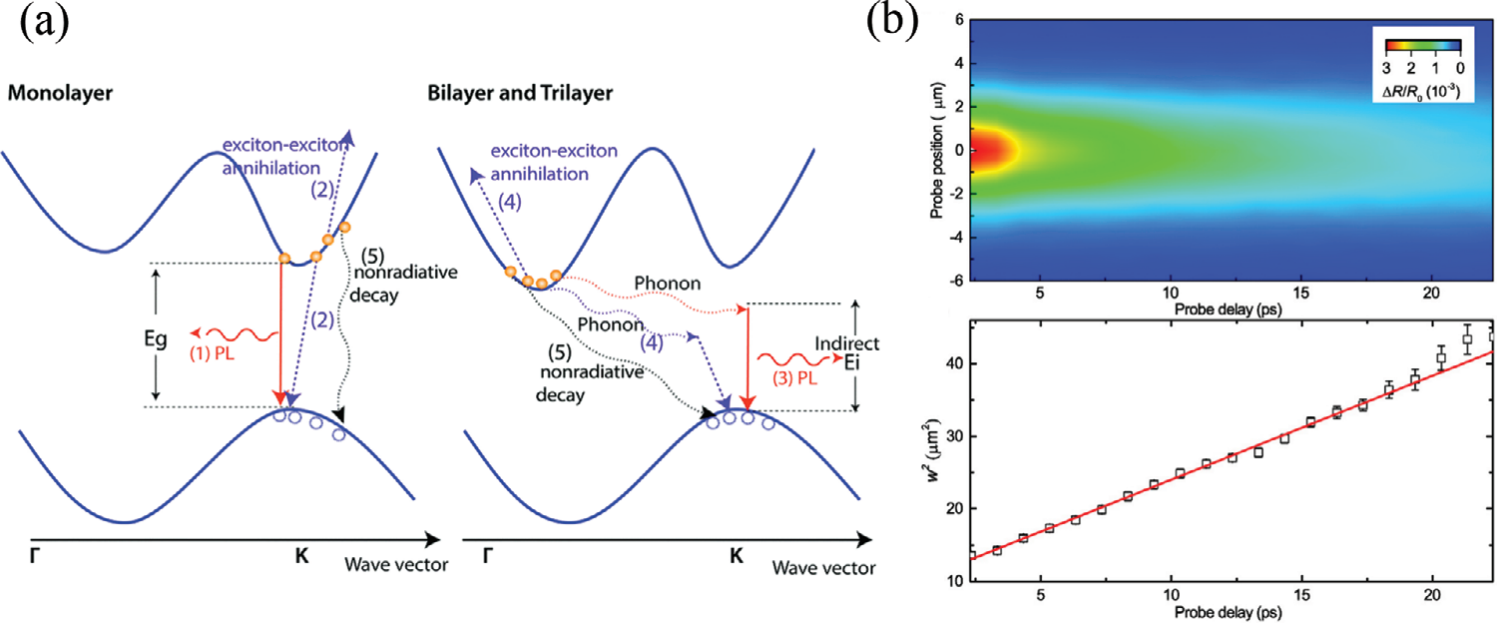
Optical inspection of carrier dynamics in 2D materials. a) Schematic representation of relaxation pathways of excitons in monolayer (left) and few‐layer (right) TMDCs. Reproduced with permission.^[^ ^194^ ^]^ Copyright 2015, The Royal Society of Chemistry. b) Top: Δ*R*/*R*_0_ plotted with respect to the probe position and probe delay along the armchair direction of BP; bottom: the correlated squared width of the spatial profile plotted with respect to the probe delay. Reproduced with permission.^[^ ^192^ ^]^ Copyright 2015, American Chemical Society.

The carrier dynamics in several 2D materials have been investigated through these ultrafast spectroscopies, including graphene,^[^ ^187^ ^]^ TMDCs,^[^ ^186^, ^188^, ^189^, ^190^ ^]^ InSe,^[^ ^191^ ^]^ and BP^[^ ^192^, ^193^ ^]^ in recent years. In these 2D materials, because of enhanced quantum confinement effects and strong coulombic interactions, several kinds of many‐body quasiparticles (e.g., excitons, trions, biexcitons) can form during the photoexcitation/relaxation process. Their formation and particular relaxation dynamics have been probed using TRPL and pump‐probe spectroscopy. Taking TMDCs as an example, several relaxation pathways have been studied for the excitons in monolayer and few‐layer TMDCs. The mechanisms, summarized schematically in **Figure** **6**a,^[^ ^194^ ^]^ include direct exciton recombination (path 1), indirect exciton recombination (path 3), exciton–exciton annihilation (EEA, paths 2 and 4), and other types of non‐radiative recombination (path 5). Notably, EEA is one of the most important modes of non‐radiative relaxation of excitons resulting from many‐body effects in TMDCs as well as BP.^[^ ^194^, ^195^, ^196^ ^]^ When EEA occurs, two excitons interact with each other and one of them is recombined rapidly and non‐radiatively. The energy from this recombination will transfer to the remaining exciton and then relax in the form of phonons. This phenomenon will dominate the recombination if the power fluence of the excitation laser is large, resulting in an increase in the non‐radiative pathway, thereby decreasing the radiative quantum yield of such 2D material‐based devices. In other words, EEA determines the maximum excitation power that can be applied to the devices. For example, even though the quantum yield of monolayer MoS_2_ improved to approximately 100% after treatment with bis(trifluoromethane)sulfonimide (TFSI), EEA still occurred when the incident power was greater than 10^‐2^ µJ cm^‐2^, such that both the carrier lifetime and the PL quantum yield decreased (Figure 6b), limiting the applications of such a device.^[^ ^185^ ^]^

The other important mode of non‐radiative recombination in 2D materials (path 5 in Figure 6a) is relaxation through the defect‐assisted Auger process; it has also been investigated using ultrafast spectroscopies. Because of strong coulombic interactions, including strong electron and hole correlations, in 2D materials, carriers are readily captured by defects and they undergo Auger scattering more effectively even at low excitation power fluence. Therefore, non‐radiative recombination in 2D materials through the Auger process will become more significant and the carrier lifetime will decrease. Notably, the mechanism of this relaxation process can be verified by investigating the temperature‐ and power fluence‐dependent carrier dynamics.^[^ ^197^ ^]^ Briefly, the carrier lifetimes in monolayer TMDCs for direct interband recombination typically range from a few tens to hundreds of picoseconds,^[^ ^186^, ^188^, ^189^, ^190^ ^]^ while the lifetimes for defect‐assisted Auger recombination are only a few picoseconds,^[^ ^190^, ^197^, ^198^ ^]^ highlighting the important role played by defects in 2D materials.

Ultrafast spectroscopies can be used not only to determine photoexcited carrier lifetimes but also, more importantly, carrier diffusion coefficients. By temporally and spatially scanning the PL intensity mapping or differential absorption/reflection signals, the squared width of the spatial profiles with respect to the delay time can be used to evaluate the diffusion coefficient according to the diffusion model (Figure 6c).^[^ ^192^ ^]^ The exciton diffusion coefficients of monolayer and bulk TMDCs^[^ ^186^, ^188^, ^189^, ^199^, ^200^ ^]^ and the anisotropic photocarrier diffusion coefficient of BP along the zigzag and armchair directions^[^ ^192^ ^]^ have been determined experimentally using the ultrafast spectroscopies. These studies can provide a deeper understanding of the behavior of the carriers in such 2D materials and, thereby, benefit the design of corresponding optoelectronic devices.

## 9 Investigating Polaritons in 2D Materials

Polaritons, which result from the collective oscillations of charge carriers, provide an opportunity to break the diffraction limit and manipulate light at the nanoscale; thus, studies of polaritons have received much interest for applications in nanophotonics. Recently, several researches have been reported that atomically thin 2D materials can support various types of polaritons. For example, plasmon polaritons in graphene and BP arise from the oscillations of free electrons;^[^ ^21^, ^201^ ^]^ exciton polaritons in TMDCs result from the wave‐like oscillations of electron/hole pairs (i.e., excitons);^[^ ^202^ ^]^ phonon polaritons in polar materials (e.g., h‐BN) originate from the atomic vibrations that are regarded as phonons.^[^ ^203^, ^204^ ^]^ The presence of polaritons and the ultrathin characteristics of 2D materials enable nanoscale light–matter interactions, making them promising materials for light detection, emission, propagation, modulation, and sensing in the fields of nanophotonics and optoelectronics. Optical inspection makes it possible to investigate the properties of polaritons in 2D materials.

**Figure 7:**
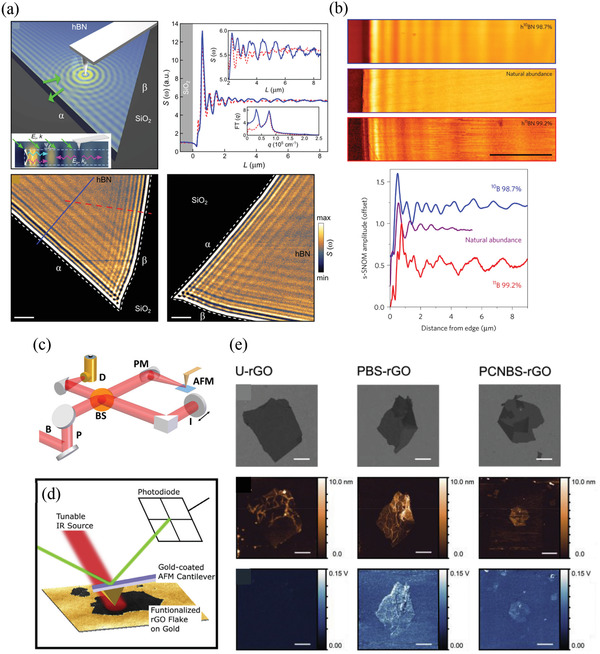
Observation of polaritons and functional groups in 2D materials. a) Upper left: Schematic representation of the experimental configuration for probing polaritons in h‐BN. Inset: Cross‐section of the tip‐excited (magenta arrows) and edge‐excited (cyan arrows; polariton fringes are also displayed as a color map) polaritons in h‐BN. Lower left: Near‐field image of h‐BN (thickness = 117 nm; *ω* = 1530 cm^‐1^; scale bar = 2 µm). Upper right: Polariton fringes scanned along directions perpendicular to the *α* (blue line) and *β* (red dashed line) edges. Inset: (top) magnified view of polariton fringe when L > 2 µm; (bottom) Fourier transform spectra of fringes. Lower right: Near‐field image of h‐BN after 90^○^ rotation. Reproduced with permission.^[^ ^203^ ^]^ Copyright 2017, American Chemical Society. b) Near‐field images (top) and amplitude profiles (bottom) collected from three h‐BN flakes having a thickness of ≈120 nm; scale bar: 5 µm. Reproduced with permission.^[^ ^206^ ^]^ Copyright 2018, Nature Publishing Group. Schematic representations of c) nano‐FTIR and d) AFM‐IR systems. e) SEM images (row 1) and AFM‐IR maps (row 2, at 1036 cm^‐1^; row 3, at 1084 cm^‐1^) of reduced graphene oxide (rGO) before (U‐rGO) and after functionalization; scale bars: 1, 2, and 1 µm, from left to right, respectively. c) Reproduced under the terms and conditions of the CC‐BY license.^[^ ^212^ ^]^ Copyright 2016, The Authors. Published by Optical Society of America. d,e) Reproduced under the terms and conditions of the CC‐BY license.^[^ ^213^ ^]^ Copyright 2018, The Authors. Published by Elsevier.

To probe the polaritons in 2D materials optically, it is necessary to overcome the wavevector mismatch between the incident light from free space and the highly confined polaritons. Several optical coupling methods fulfilling this requirement have been proposed to excite the polaritons in 2D materials and, thereby, allow investigation of the polariton behavior; for example, coupling with diffraction gratings or nanostructures, prisms in attenuated total reflection (ATR), and AFM tips in s‐SNOM. Here, our discussion focuses on the use of s‐SNOM for the probing of polaritons, because the other methods can possibly damage the 2D material samples. s‐SNOM is an optical inspection method based on AFM; it has been applied to investigate the behavior of polaritons in graphene,^[^ ^21^ ^]^ TMDCs,^[^ ^202^ ^]^ h‐BN,^[^ ^203^, ^204^ ^]^ and BP.^[^ ^201^ ^]^ When monochromatic light from a laser (typically a quantum cascade laser) is applied incident to the AFM tip coated with a metal at an oblique angle, a portion of the light will be scattered back by the tip and some will be coupled to polariton waves in the 2D material, propagating radially outward from the tip until the polariton waves reach the sample edges or defects (as illustrated in **Figure** **7**a).^[^ ^203^ ^]^ For graphene, h‐BN, and BP, the edges or defects act as reflectors, such that the polariton waves are reflected back to the tip and scattered again. The directly back‐scattered and reflected polariton waves experience interference. By scanning the tip toward the edge, a standing wave oscillation fringe will be observed, representing the interference of the polaritons. A similar interference fringe can be observed for TMDCs, except that this fringe results from interference between the directly back‐scattered and edge‐ or defect‐scattered light, because the edges or defects act as scatterers for TMDCs polaritons.^[^ ^202^ ^]^ This different interference behavior in TMDCs results from the characteristics of their polaritons being distinct from those of other 2D materials.^[^ ^202^ ^]^ Notably, when the AFM tip is close to the sample edge, the laser light beam will possibly also illuminate the edge, thereby exciting the polariton wave. In this case, the edge‐excited polariton wave will contribute additionally to the interference, and this effect should be taken into account. Briefly, investigations of polaritons in 2D materials can be performed by resolving the interference fringes scanned by the s‐SNOM system. By this means, the wavelength of the polariton *λ*_p_, the confinement ratio *λ*_0_/*λ*_p_ (*λ*_0_ is the wavelength of the incident laser light), and the frequency (*ω*)‐momenta (*k*) dispersion curve of the 2D material can be obtained. Figure 7a displays an example of the inspection of a phonon polariton in h‐BN through s‐SNOM.^[^ ^203^ ^]^ h‐BN is a natural hyperbolic material for which the in‐plane and out‐of‐plane real parts of the dielectric constants have opposite signs, thereby supporting hyperbolic phonon polaritons. These special polaritons exhibit ultrahigh confinement and low loss characteristics, making h‐BN a promising material for use in metamaterials and waveguides.^[^ ^205^ ^]^

s‐SNOM has been used to investigate the behavior of polaritons in various 2D materials, revealing a strong dependence on their structural properties. For example, the plasmon polariton wavelength (*λ*_p_) at *ω* = 892 cm^‐1^ (corresponding to a free space wavelength of 11.2 µm) decreased upon increasing the electron concentration in graphene.^[^ ^21^ ^]^ On the other hand, the phonon polaritons in natural h‐BN decay rapidly because the boron isotopes in natural h‐BN comprise approximately 80% ^11^B and 20% ^10^B and, therefore, 10% of the atomic mass of boron can act as scattering centers for the phonon polaritons. By using isotopically enriched boron powder, samples of h‐BN can be grown with nearly pure ^11^B or ^10^B, and these materials can support phonon polaritons with longer propagation lengths (Figure 7b).^[^ ^206^ ^]^ Furthermore, the dispersion of phonon polaritons in h‐BN is layer‐number dependent,^[^ ^204^, ^207^ ^]^ with monolayer or bilayer h‐BN being able to support polaritons with a significant confinement accompanied by long propagation lengths.

## 10 Identifying Functional Groups in 2D Materials

Chemical functionalization of 2D materials is a promising method for expanding their applicability in various electronic and optoelectronic devices. Through covalent or noncovalent surface modification, the physical and chemical properties of a 2D material can be readily tuned to achieve a required performance. For example, a bandgap can be induced in graphene, and its energy tuned continuously, when covalently bonding hydrogen or fluorine atoms;^[^ ^208^ ^]^ the quantum yields of monolayer MoS_2_ and WS_2_ can both be improved to approximately 100% after treatment with TFSI molecules;^[^ ^209^ ^]^ surface modification of BP with fluorine atoms can provide passivation layers that prevent it from degradation when exposed to O_2_ and humidity;^[^ ^210^ ^]^ the electrical and optical properties of several 2D materials (e.g., graphene, TMDCs, BP) can be adjusted through chemical doping, by adsorbing molecules that introduce charge transfer, as discussed above.^[^ ^167^ ^]^

Although covalent and noncovalent bonding of chemical species can adjust the properties of 2D materials to achieve a required performance, these added molecules or atoms can also function as defects if they result in unwanted properties—especially for BP, which is very sensitive to O_2_ and humidity. In either case, non‐destructive methods for identification of the modifying atoms and functional groups in 2D materials would facilitate monitoring of their physical properties. Although Raman spectroscopy is a useful tool for probing the lattice vibrations in 2D materials, such that much structural information can be obtained from their Raman spectra, the Raman polarizability selection rules mean that several functional groups will be spectroscopically weak or Raman‐inactive. When these functional groups are attached to a 2D material, they might appear in Raman spectra only through their effects on the carrier doping or disorder of the original structure. Fortunately, functional groups that are Raman‐inactive are usually IR‐active; thus, they can be identified using IR spectroscopy—in particular, FTIR spectroscopy. Because functional groups absorb IR light at specific wavelengths, the absorption peaks in IR spectra of 2D materials can provide information about the types of bonded functional groups. Accordingly, several functional groups in 2D materials have been characterized by analyzing their FTIR spectra, including the hydroxyl (OH) and amino (NH_2_) groups formed at the edges of h‐BN when exposed to moisture^[^ ^211^ ^]^ and the C–F and P–F covalent bonds formed during the fluorination of graphene and BP, respectively.^[^ ^208^, ^210^ ^]^ Notably, the fluorination of BP results in no significant changes in its Raman spectrum, whereas its FTIR spectrum exhibits several characteristic absorption peaks associated with P–F bond vibrations, revealing the complementarity of Raman and FTIR spectroscopy.^[^ ^210^ ^]^

Two techniques combining AFM and IR spectroscopy have been applied recently to identify the functional groups in 2D materials with nanoscale spatial resolution: nano‐FTIR spectroscopy and AFM‐IR spectroscopy, which are distinguished by their working principles. Nano‐FTIR spectroscopy is somewhat similar to s‐SNOM, except that a broadband IR light source or tunable quantum cascade laser is used (Figure 7c).^[^ ^212^ ^]^ In contrast, AFM‐IR spectroscopy measures the absorption of incident IR light by converting the thermal expansion of samples (resulting from the light absorption) to mechanical motion of the cantilever during AFM operation in the tapping mode (Figure 7d).^[^ ^213^ ^]^ Using nano‐FTIR and AFM‐IR spectroscopic techniques, the functional groups in 2D materials can be identified and quantified with high resolution (<20 nm), determined by the size of the AFM tip. Figure 7e presents an example of the nanoscale identification and mapping of the physically adsorbed sodium 4‐(4,5a1‐dihydropyrene‐1‐yl)butane‐1‐sulfonate (PBS) and sodium 4‐(7‐cyano‐4,5a1‐dihydropyrene‐1‐yl)butane‐1‐sulfonate (PCNBS) on reduced graphene oxide (rGO).^[^ ^213^ ^]^

## 11 Probing Lattice Spacings in 2D Materials

Lattice spacing is the distance between the planes of atoms in a crystalline material. Therefore, probing lattice spacing can provide information about the arrangement of atoms or molecules in a crystal, assisting studies of its physical or chemical properties. Typically, lattice spacings [of approximately a few Å] in 2D materials have been studied by using the techniques of X‐ray scattering, diffraction, and reflectivity. For example, the out‐of‐plane lattice spacings (i.e., c‐axis lattice parameters) after intercalation of hydrogen or oxygen atoms in graphene on silicon carbide (SiC)^[^ ^214^ ^]^ and those resulting from electrochemical lithiation of mechanically exfoliated graphene flakes^[^ ^215^ ^]^ have been investigated using X‐ray reflectivity (XRR). In addition, the in‐plane arrangement of carbon atoms in graphene has been investigated using grazing incidence X‐ray diffraction (GIXD).^[^ ^215^ ^]^ Notably, the structural dynamics of 2D materials can also be investigated by combining pump‐probe spectroscopy with ultrafast X‐ray spectroscopy to provide the probing light. Through ultrafast X‐ray spectroscopy, both the in‐plane and out‐of‐plane lattice vibrations of monolayer WSe_2_ on Al_2_O_3_ have been studied under the incidence of a pump light pulse having a wavelength of 650 nm (close to the B‐exciton absorption peak).^[^ ^216^ ^]^ The experimental results revealed that the absorbed photon energy, which was near the excitonic absorption peak of WSe_2_, was coupled preferably to the in‐plane lattice vibrations, rather than the out‐of‐plane vibrations. Accordingly, X‐ray spectroscopy is indeed a useful tool for probing the lattice spacings in 2D materials.

## 12 Optical Inspection of vdW Integration of 2D Materials

vdW integrations of 2D materials comprise various monolayer or few‐layer 2D materials stacked with each other using transfer techniques and stabilized by weak vdW forces.^[^ ^40^ ^]^ When one 2D material is heterogeneously integrated with another, forming a so‐called 2D vdW heterostructure, fascinating properties, quite different from the intrinsic properties of the individual 2D materials, might possibly emerge as a result of the vdW interactions. Accordingly, the vdW integrations of 2D materials have broad applicability in electronics, photonics, and optoelectronics.^[^ ^40^, ^41^ ^]^ To ensure the required performance of 2D vdW heterostructures in devices, it would be critical to be able to precisely probe the properties of the 2D materials before and after their assembly. So far, we have reviewed several optical inspection techniques for characterizing the structural properties (e.g., layer number, defect density, strain, doping) of various 2D materials prepared through mechanical exfoliation or CVD (i.e., the 2D materials prior to assembly). Here, we highlight some of the key interactions between different types of 2D materials after vdW integrations, and discuss the optical inspection techniques used to probe them. Our goal is to provide some guideline for characterizing the vdW integrations of 2D materials rapidly and non‐destructively when tuning the performances of corresponding 2D material‐based devices.

Before discussing the interactions between different types of 2D materials in their vdW integrations, we need to identify their basic heterostructures. The most important structural characteristics of a vdW heterostructure are the location of the integration area and its composition (type and layer number), especially when it is prepared by transferring mechanically exfoliated 2D flakes (usually small in terms of their lateral size) on top of another one, and so on, for integration. To rapidly locate the integration area of a vdW heterostructure, the mostly effective strategy is to prepare it on the surface of a substrate that provides high optical contrast for visualizing 2D materials.^[^ ^55^ ^]^ By optimizing the design of the substrate with enhanced visibility, each 2D material component and its integration area can be readily located and visualized. Although several vdW heterostructures (e.g., graphene/h‐BN, graphene/MoS_2_, graphene/h‐BN/MoS_2_, graphene/MoS_2_/h‐BN) have been visualized on SiO_2_/Si substrates,^[^ ^217^ ^]^ other substrates have yet to be investigated. Accordingly, the optimal structures for visualizing 2D vdW heterostructures remain to be identified.

When preparing 2D vdW integrations, especially those comprising more than two types of 2D materials (i.e., vdW heterostructures with high complexity), identification of their compositions is necessary to ensure that the desired heterostructures are achieved. For determining the types and layer numbers of the individual 2D materials in vdW heterostructures, the most suitable techniques for optical inspection are optical spectroscopy, Raman spectroscopy, and PL spectroscopy, because each 2D material possesses its own layer‐dependent optical characteristics that can be probed by these tools, as discussed above.^[^ ^62^, ^71^, ^74^, ^92^ ^]^ For example, reflectance spectra in the visible regime have been investigated theoretically and experimentally for vdW integrations comprising graphene and h‐BN on SiO_2_/Si substrates having SiO_2_ layers of various thicknesses.^[^ ^218^ ^]^ Because graphene is an absorptive material, it affects a dip in the reflectance spectra (in other words, causes a reflectance minimum), with the absolute reflectance decreasing and the wavelength red‐shifting when graphene is present. On the other hand, h‐BN is transparent; therefore, the effect of h‐BN on a SiO_2_/Si substrate is dominated by wavelength shifts only, arising from the additional phase difference attributed to h‐BN. Accordingly, the reflectance minima for graphene and h‐BN vdW integrations can be regarded as fingerprints for distinguishing the layer numbers of such vdW heterostructures.^[^ ^218^ ^]^

Raman and PL spectroscopies have also been used to characterize the types and layer numbers of the individual 2D materials in vdW integrations. For example, the Raman spectra of a MoS_2_/WS_2_ heterostructure, comprising a MoS_2_ flake transferred onto a WS_2_ flake, have been measured to confirm that both of the components were monolayers, identified by the fact that the Raman spectra appeared to be the sum of Raman spectra of the respective MoS_2_ and WS_2_ monolayers.^[^ ^219^ ^]^ Furthermore, the PL spectrum of a MoSe_2_/WSe_2_ heterostructure featured two characteristic PL peaks at 1.65 and 1.57 eV, coincident with the PL peaks of monolayer MoSe_2_ and WSe_2_, respectively.^[^ ^42^ ^]^ As a result, Raman and PL spectroscopies can indeed be applied to identify the compositions of 2D vdW heterostructures.

Optical inspection techniques have also been used to probe the vdW interactions between different types of 2D materials. The most commonly studied heterostructures of 2D vdW integrations have been those assembled with h‐BN, because it has an atomically smooth surface with no dangling bonds or charge traps that could possibly influence the performance of the integrated 2D materials.^[^ ^220^, ^221^, ^222^, ^223^ ^]^ Accordingly, h‐BN has been used as a substrate for various 2D materials, allowing them to exhibit their intrinsic characteristics, thanks to the fact that the h‐BN substrate can protect them from being disturbed by a charged surface state or surface roughness. Furthermore, h‐BN has also been used as an encapsulation layer for 2D materials, preventing them from being exposed to O_2_ and moisture. With the capping of h‐BN, the optical and electrical properties of graphene,^[^ ^220^ ^]^ TMDCs,^[^ ^222^ ^]^ InSe and GaSe,^[^ ^221^ ^]^ and BP^[^ ^223^ ^]^ have been greatly enhanced relative to those on conventional SiO_2_/Si substrates, as revealed using Raman and PL spectroscopies. Briefly, for h‐BN‐encapsulated graphene and BP,^[^ ^220^, ^223^ ^]^ the narrower FWHMs of their characteristic Raman peaks suggested superior quality;^[^ ^106^ ^]^ the narrower FWHMs of the excitonic PL peaks of TMDCs^[^ ^222^ ^]^ meant that h‐BN could effectively suppress fluctuations and the introduction of defects arising from the SiO_2_ surface;^[^ ^121^ ^]^ and the enhanced PL intensities and lower PL decay rates of encapsulated InSe and GaSe^[^ ^221^ ^]^ demonstrated their high quality and long‐term stability.^[^ ^120^ ^]^

**Figure 8:**
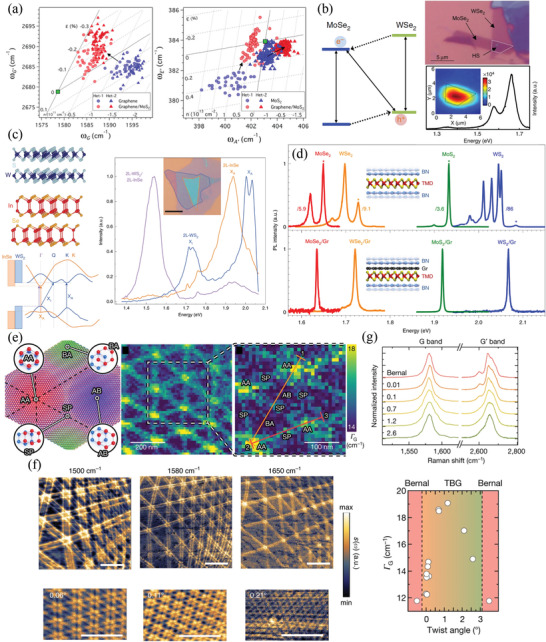
Optical inspection of vdW integrations of 2D materials. a) Correlation analysis of Raman spectral peak positions of graphene (*ω*_G_, G band; *ω*_G’_, 2D band; left panel) and MoS_2_ (*ω*_A’_, A_1g_ mode; *ω*_E’_, $E_{2g}^{1}$ mode; right panel) before (blue) and after (red) vdW integration. Reproduced with permission.^[^ ^224^ ^]^ Copyright 2019, American Physical Society. b) Left: Schematic representation of the formation of an interlayer exciton in a MoSe_2_/WSe_2_ heterostructure; upper right: optical image of MoSe_2_/WSe_2_ heterostructure; lower right: PL spectra obtained from the MoSe_2_/WSe_2_ heterostructure region (inset: mapping of PL intensity at energies from 1.273 to 1.400 eV). Reproduced with permission.^[^ ^42^ ^]^ Copyright 2015, Nature Publishing Group. c) Right: PL spectra of 2L‐WS_2_ (blue), 2L‐InSe (orange), and the 2L‐WS_2_/2L‐InSe heterostructure (purple); inset: optical image of 2L‐WS_2_/2L‐InSe heterostructure (scale bar: 10 µm). Left: Schematic representations of the crystal structures of 2L‐WS_2_ and 2L‐InSe (upper left); band structure (lower left); blue, orange, and purple arrows in the band structure highlight the corresponding transitions. Reproduced with permission.^[^ ^229^ ^]^ Copyright 2020, Nature Publishing Group. d) Normalized PL spectra of four TMDCs without (top) and with (bottom) the integration of graphene; measurements were performed at temperatures below 20 K. Reproduced with permission.^[^ ^230^ ^]^ Copyright 2020, Nature Publishing Group. e) Schematic representation (left) and mapping of the FWHM of the Raman G band (middle and right) from a TBG sample, recorded using nano‐Raman spectroscopy; twist angle: 0.09^○^. Reproduced with permission.^[^ ^231^ ^]^ Copyright 2021, Nature Publishing Group. f) Top: s‐SNOM images of TBG moiré superlattices, recorded at the same positions but with different excitation frequencies; twist angle: 0.05^○^. Bottom: s‐SNOM images of TBG with different twist angles, recorded at 1560 cm^–1^; scale bars: 500 nm. Reproduced under the terms and conditions of the CC‐BY license.^[^ ^235^ ^]^ Copyright 2020, The Authors. Published by Nature Publishing Group. g) FWHM of the Raman G band from TBG, plotted with respect to twist angle, measured using conventional Raman spectroscopy. Reproduced with permission.^[^ ^231^ ^]^ Copyright 2021, Nature Publishing Group.

As noted in previous sections, Raman and PL spectroscopies are powerful tools for probing not only the defect densities (i.e., structural quality) but also the strain and doping of 2D materials; accordingly, they can also be used to evaluate the degrees of strain and doping in vdW heterostructures. **Figure** **8**a provides examples of the identification of strain and doping in graphene and MoS_2_, respectively, through investigations of the peak positions of their Raman modes (G and 2D bands for graphene; A_1g_ and $E_{2g}^{1}$ modes for MoS_2_) in two different graphene/MoS_2_ heterostructures, based on the vector model described in Figure 5d,e.^[^ ^181^, ^224^ ^]^ According to the direction of the shift in the peak positions of the graphene G and 2D bands after vdW integration, compressive strain and electron doping had been introduced into the graphene; on the other hand, compressive strain and hole doping had been introduced into the MoS_2_, as characterized by the peak shifts of its A_1g_ and $E_{2g}^{1}$ modes.^[^ ^224^ ^]^ Combining these two features reveals that electrons had been transferred from the MoS_2_ to the graphene upon the assembly of the vdW heterostructure. PL spectra of the MoS_2_ before and after assembling these two heterostructures (not shown here) also suggested the effects of compressive strain and hole doping in the MoS_2_, matching well the Raman spectral characteristics in Figure 8a.^[^ ^224^ ^]^

One emerging group of vdW integrations is based on band alignment of individual semiconducting 2D materials, thereby allowing interlayer transitions or transfers (depending on the type of band alignment, as discussed below) between them, such that these vdW heterostructures are potentially useful in field of optoelectronics. Typically, two types of band alignment are possible, depending on the work functions of the component 2D materials. Type‐I band alignment exists when one material having a narrower bandgap is integrated with another having a wider bandgap, and both the conduction band minimum (CBM) and the valence band maximum (VBM) are located within the material having the wider bandgap. The photo‐excited electrons and holes in the wide‐band‐gap material will both transfer immediately to the narrow‐band‐gap material, with quantum confinement effects resulting in increased radiative recombination—favorable characteristics for applications in light‐emitting devices.^[^ ^225^ ^]^ In the type‐II band alignment, on the other hand, the CBM and VBM are located in different 2D materials. As a result, when carriers are excited in the material having the conduction band (valence band) with higher (lower) energy level, electrons (holes) will transfer to the other material (i.e., an interlayer transition will occur), followed by formation of interlayer excitons (Figure 8b).^[^ ^42^ ^]^ Because the electrons and holes are spatially separated in different materials, the type‐II band alignment will likely benefit applications in photovoltaics and photodetectors.^[^ ^225^ ^]^ Furthermore, a small interlayer transition energy, determined by the difference between the CBM and VBM in such a heterostructure, allows the detection of photon energy below the cutoffs (bandgap) of the respective 2D materials.^[^ ^226^ ^]^ According to the main features of type‐I and type‐II band alignments, the optical and electrical properties of vdW heterostructures can be readily tuned to ensure the desired performance of their corresponding devices; therefore, techniques for optical inspection of their properties can be very useful.

Most combinations of semiconducting 2D materials feature a type‐II band alignment; thus, our discussion here focuses mainly on the optical inspection of type‐II heterostructures. The interlayer transition energies and charge transfer dynamics of type‐II heterostructures have been characterized using PL spectroscopy and pump‐probe spectroscopy, respectively.^[^ ^42^, ^219^, ^227^, ^228^ ^]^ For example, the PL spectrum of a MoSe_2_/WSe_2_ heterostructure featured three peaks located at 1.65, 1.57, and 1.35 eV (Figure 8b).^[^ ^42^ ^]^ The first two peaks match the PL photon energies of the monolayer MoSe_2_ and WSe_2_, respectively. The peak at 1.35 eV is lower than their bandgap energies; therefore, it is recognized as the interlayer exciton PL corresponding to the interlayer transition energy, confirmed by the fact that this peak appears only in the heterostructure region through PL mapping (inset to Figure 8b). We provide two examples of the investigation of carrier dynamics through pump‐probe spectroscopy: the hole transfer from MoS_2_ to WS_2_ was found to occur within approximately 50 fs^[^ ^219^ ^]^ and the electron transfer from BP to MoS_2_ was identified to occur within approximately 5 ps.^[^ ^227^ ^]^

In type‐II heterostructures, the electrons and holes tend to accumulate in the different 2D materials to form interlayer excitons. This phenomenon suggests the feasibility of applying such heterostructures as light‐emitting devices with tunable spectral wavelengths determined by their interlayer transition energies. Nevertheless, mismatched momentum of the electron/hole pair for interlayer recombination usually limits the light emission efficiency of such a heterostructure, exemplified by the low intensity of the interlayer exciton PL peak at 1.35 eV in Figure 8b. To increase the interlayer emission, one strategy is to form a 2D vdW integration that favors a direct interlayer transition by choosing 2D materials having the CBM located at the Γ point (center) of the Brillouin zone in one material and the VBM located at the same point in the other.^[^ ^229^ ^]^ More specifically, because the Γ point is the center corresponding to zero momentum (*k* = 0), all materials coincide at this point regardless of their lattice constants and crystalline alignment, such that a direct interlayer transition is achieved more readily once the CBM and VBM in the respective 2D materials are located at the Γ point. This strategy can be verified by observing the PL spectra of bilayer WS_2_ (2L‐WS_2_), bilayer InSe (2L‐InSe), and the 2L‐WS_2_/2L‐InSe heterostructures (Figure 8c)^[^ ^229^ ^]^ The PL peak located at 1.55 eV (as represented by the purple curve), with photon energy lower than that of the constituent materials, corresponds to the direct interlayer transition of the heterostructure (as represented by the purple arrow in the schematic representation of electronic band structure). These PL spectra confirm the successful practical implementation of the design of heterostructures following the proposed strategy; such characterization should broaden the applicability of vdW integrations.

A filtering effect has been found recently in graphene/TMDC vdW heterostructures, probed and investigated using PL and TRPL spectroscopies.^[^ ^230^ ^]^ The filtering effect involved the graphene completely neutralizing the TMDCs when stacked upon them, such that only a neutral exciton emission appeared in their PL spectra (Figure 8d).^[^ ^230^ ^]^ In general, the characteristic PL peak intensity of TMDCs after integration with graphene would decrease, because the photoexcited charge carriers (with lifetimes on the range of nanoseconds) could transfer immediately to graphene (within a few picoseconds); this process is known as PL quenching. When the graphene/TMDC vdW heterostructures were investigated at low temperature, the radiative lifetime of the neutral excitons of the TMDCs was significantly shortened, while that of the charged excitons (i.e., trions) remained long, and both of them could be probed using TRPL. When the lifetime of the neutral excitons was close to the transfer time, radiative recombination occurred prior to charge transfer to graphene, so that the PL intensity was minimally influenced. In contrast, the trion emission was effectively quenched by graphene because the charge transfer was fast. As a result, the PL spectra of graphene/TMDC vdW heterostructures reveal the filtering effect, and feature only the neutral exciton emission with a narrow FWHM.^[^ ^230^ ^]^ With the help of optical inspection, this filtering effect was revealed to be an important vdW interaction in 2D vdW integrations.

So far, we have reviewed techniques of optical inspection for characterizing the vdW interactions in 2D vdW integrations consisting of heterogeneous materials. Surprisingly, some hetero‐bilayer vdW integrations, comprising homogeneous materials, also possess novel properties when one layer is intentionally stacked on the other with a twist angle, forming a periodic pattern known as a “moiré superlattice.”^[^ ^231^, ^232^, ^233^ ^]^ For example, when two graphene layers are stacked with a small twist angle, a moiré pattern is formed, comprising alternating AB and BA triangular stacking domains with shear solitons (SP stacking) as domain walls, with the vertices of the triangular area being AA‐stacking regions (Figure 8e).^[^ ^231^ ^]^ With the presence of the superlattice, the electronic band structure of the twist bilayer graphene (TBG) will be modulated as a result of interlayer coupling, forming a superlattice bandgap and an enhanced density of states. As a result, TBG can be used as mid‐infrared photodetector in the wavelength range from 5 to 12 µm, with a maximum photoresponse when the twist angle is 1.81^○^.^[^ ^234^ ^]^ Interestingly, TBG also possesses superconductivity at a temperature of 1.7 K with a twist angle of 1.1^○^, the so‐called “magic angle.”^[^ ^43^ ^]^ In contrast, the indirect bandgaps in twist bilayer TMDCs (e.g., bilayer MoS_2_) can be tuned continuously by varying the twist angle.^[^ ^232^ ^]^ Because the properties of twist bilayer systems are highly dependent on the twist angle, optical inspection becomes a rapid and non‐destructive means of monitoring and controlling their properties.

The optical inspection of twist bilayers has mainly focused on direct imaging of TBG moiré superlattices using nano‐FTIR^[^ ^235^ ^]^ or nano‐Raman^[^ ^231^ ^]^ spectroscopy. Nano‐Raman spectroscopy is a technique combining s‐SNOM and Raman spectroscopies; it can achieve Raman spectral signals with nanoscale spatial resolution. In nano‐FTIR, the AB/BA domains and their domain walls (SP) in TBG provide different IR signals; thus, the moiré superlattice can be imaged by mapping the IR signals.^[^ ^233^ ^]^ Similarly, because the FWHM of the TBG Raman G band from the AA‐stacking region is the largest, while that from the AB‐/BA‐stacking is the smallest, these bands can be used as characteristics for constructing the image of the moiré superlattice (Figure 8e).^[^ ^231^ ^]^

Visualization of the TBG moiré superlattice can also be performed by using s‐SNOM to map the polaritons.^[^ ^236^ ^]^ When the plasmonic polaritons in TBG are excited through illumination of the AFM tip, they propagate radially outward from the tip, and are be reflected back by the domain walls (SP stacking). As a result, an interference fringe with double‐line features, parallel to the domain wall, will form; it can be regarded as a direct image of the TBG moiré superlattice.^[^ ^236^ ^]^ Because this imaging technique requires doping in TBG, an alternative approach that probes the phonon polariton in the h‐BN encapsulation layer has also been proposed; Figure 8f displays images of the resulting TBG moiré superlattices.^[^ ^235^ ^]^ Notably, the spacing of the double‐line decreased upon increasing the polariton frequency, because the wavelength of the phonon polariton decreased, providing better resolution when visualizing the TBG.

In studies visualizing the TBG moiré superlattice, the twist angle can be derived from its relationship to the superlattice unit cell area (*A*), expressed by following equation:^[^ ^234^ ^]^

(8) $$A=\frac{\sqrt{3}}{2}{\left(\frac{a}{2\sin\frac{\vartheta }{2}}\right)}^{2}$$

where *a* is the lattice constant of graphene (*a* = 0.246 nm) and *θ* is the twist angle in TBG. According to this equation, the twist angle of TBG can be identified (Figure 8f), but it will be difficult to characterize a twist angle of greater than 1^○^ because the area of the superlattice unit cell will be too small to resolve (the spatial resolutions of nano‐FTIR and nano‐Raman spectroscopy are on the order of a few tens of nanometers). To probe twist angles greater than 1^○^, especially for angles around the magic angle of 1.1^○^, the FWHM of the TBG G bands, measured using conventional Raman spectroscopy, can be considered as a fingerprint of the twist angle (Figure 8g).^[^ ^231^ ^]^ In addition, conventional Raman spectroscopy has also been used to identify the twist angles of bilayer TMDCs by probing the peak shifts of their Raman A_1g_ modes.^[^ ^232^ ^]^

## 13 Wafer‐Scale Optical Inspection of 2D Materials

**Figure 9:**
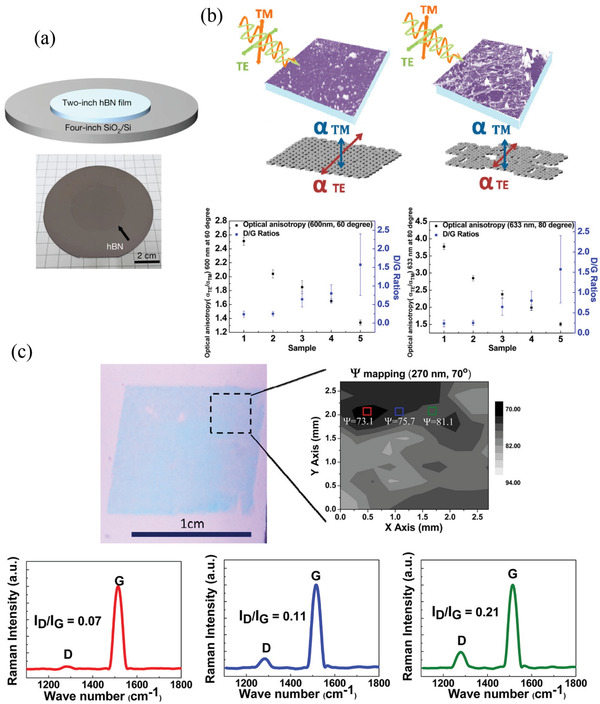
Wafer‐scale growth of 2D materials and their optical inspection. a) Schematic representation (top) and photograph (bottom) of the wafer‐scale h‐BN transferred onto a 4 in. SiO_2_/Si substrate. Reproduced with permission.^[^ ^44^ ^]^ Copyright 2020, Nature Publishing Group. b) Top: Schematic representations of the different absorption behaviors of monolayer graphene under different polarizations; bottom: correlations between the optical anisotropy and the intensity ratio of the graphene Raman D and G bands of various graphene samples. Reproduced with permission.^[^ ^241^ ^]^ Copyright 2013, American Chemical Society. c) Photograph of a monolayer graphene sample and mapping of the ellipsometric parameter, Ψ, obtained from the dashed area; corresponding Raman spectra measured from the three colored rectangles are also displayed (bottom). Reproduced with permission.^[^ ^242^ ^]^ Copyright 2014, American Chemical Society.

Major efforts have been devoted to the synthesis of 2D materials with high quality over a large area, to overcome the main disadvantage of the Scotch tape method, which can provide 2D flakes with only small areas, thereby, restricting the applications of such materials in practical devices. Recently, researches have shown that the wafer‐scale single‐crystal h‐BN films have been sucessfully grown on molten gold surface through the formation of self‐collimated h‐BN grains^[^ ^237^ ^]^ and on a bulk Cu(110) foil which was obtained by annealing an industrial Cu foil.^[^ ^238^ ^]^ In addition, an indicative study was reported for the growth of a two‐inch‐wafer‐sized single‐crystal h‐BN monolayer on a Cu(111) thin film/*c*‐plane sapphire substrate (**Figure** **9**a),^[^ ^44^ ^]^ suggesting the feasibility of growing other 2D materials on the wafer scale. To facilitate the development of wafer‐scale growth techniques for various 2D materials, in situ monitoring of the global properties of the synthesized 2D materials, especially their structural quality, would be critical when adjusting the growth parameters. Optical inspection techniques allow materials to be characterized non‐destructively; therefore, they are potential candidates for in situ inspection of the global properties of 2D materials. Raman and PL spectroscopies are two particularly powerful tools for characterizing 2D materials, as we have demonstrated repeatedly in this Review. Although these techniques provide abundant information regarding the layer numbers, defect densities, and degrees of doping of 2D materials, the small areas of their measured spots limit their applicability for wafer‐scale inspection. Optical spectroscopy and spectroscopic ellipsometry are alternative techniques for probing 2D materials on the wafer‐scale, due to their relatively larger spot sizes. Optical spectroscopy is a technique that reveals the fundamental optical properties of a sample by measuring its reflectance, transmittance, and absorptance spectra. Spectroscopic ellipsometry collects and analyzes the transverse magnetic (TM)‐ and transverse electric (TE)‐polarized reflected light, allowing the amplitude ratio (Ψ) and phase difference (Δ) between their reflection coefficients to be determined as the main ellipsometric parameters. These two optical inspection tools have been applied mainly to counting the layer numbers^[^ ^15^, ^62^, ^63^, ^64^ ^]^ and measuring the optical constants^[^ ^16^, ^239^ ^]^ of 2D materials. Although refractive indices (*n*) and extinction coefficients (*k*) have been considered as good indicators of structural quality,^[^ ^240^ ^]^ they are generally derived using fitting procedures, meaning that correct results can be obtained only when choosing appropriate and adequate fitting parameters. Accordingly, it would be preferable to have another indicator of structural quality that can be inspected using optical spectroscopy or spectroscopic ellipsometry.

Optical anisotropy has been proposed as a quality factor for evaluating the structural quality of graphene.^[^ ^241^ ^]^ Because of the planar nature of graphene, its absorption of polarized light depends significantly on the angle of incidence. When TE‐polarized light is applied incident to graphene, the direction of the electric field is perpendicular to the incident plane and parallel to the plane of graphene surface; as a result, the absorption coefficient (*α*_TE_) should remain almost constant upon increasing the incident angle. On the other hand, the angle between the electric field of TM‐polarized light, which is parallel to the incident plane, and the plane of the graphene surface will increase upon increasing the incident angle; as a result, less incident light will be absorbed by the graphene, decreasing the absorption coefficient of the TM‐polarized light (*α*_TM_). The ratio of the absorption coefficients of the TE‐ and TM‐polarized light at various angles of incidence is defined as the optical anisotropy of graphene. The optical anisotropy of light absorption, which can be measured using optical spectroscopy, is strongly related to the planar structure of graphene. If vacancies, wrinkles, or cracks are present, the in‐plane light absorption (*α*_TE_) would decrease, such that the optical anisotropy would decrease (Figure 9b). Therefore, optical anisotropy can act as an indicator of the structural imperfections in graphene; indeed, the correlation between the optical anisotropy and the structural quality of graphene samples has been investigated experimentally and theoretically.^[^ ^241^ ^]^ Notably, optical anisotropy has been verified as a structural factor by correlating it to the values and deviations of the intensity ratio of the graphene Raman D and G bands (Figure 9b).^[^ ^241^ ^]^ Accordingly, optical anisotropy, determined directly using optical spectroscopy, can indeed be a quality factor for the structure of graphene.

When optically characterizing the structural properties of wafer‐scale 2D materials, spectroscopic ellipsometry would presumably be a more powerful tool than conventional optical spectroscopy because of its highly reproducible signals and better resolution, based on the measuring the amplitude ratio of reflected light rather than the absolute intensities of reflected and transmitted light. Indeed, it has also been applied to study the correlation between the optical anisotropy of graphene and its structural quality.^[^ ^242^ ^]^ In spectroscopic ellipsometry, the two main ellipsometric parameters, Ψ and Δ, are determined by analyzing the reflected TM‐ and TE‐polarized light, described by

(9) $$\frac{r_{\mathrm{TM}}}{r_{\mathrm{TE}}}=\tan\Psi \times e^{i\Delta }$$

where *r*_TM_ and *r*_TE_ are the complex reflection coefficients of the TM‐ and TE‐polarized light, respectively; tan Ψ is the amplitude ratio of *r*_TM_ and *r*_TE_; and Δ is the phase difference between these two types of light. When using spectroscopic ellipsometry to characterize graphene, one of the ellipsometric parameters, Ψ, is strongly influenced by its optical anisotropy, as determined both experimentally and theoretically.^[^ ^242^ ^]^ The structural quality of graphene can, therefore, be evaluated by using spectroscopic ellipsometry to monitor the value of Ψ directly; this method is easier than measuring the optical anisotropy from the absorption of light with different types of polarization. The practicality of large‐area mapping confirms that this ellipsometry‐based method is very convenient and useful for the rapid identification of graphene (Figure 9c).^[^ ^242^ ^]^

Spectroscopic ellipsometry has been applied to characterize wafer‐scale 2D materials, mainly to determine optical constants and to monitor the uniformity in thickness over the wafer‐size regions during the growth of wafer‐scale 2D materials.^[^ ^240^, ^243^ ^]^ With the help of optical anisotropy, wafer‐scale characterization of the structural quality of 2D materials is believed to be possible, although this concept has only verified been for graphene. Because of the planar nature of 2D materials, we believe that various other 2D materials should possess optical anisotropy and that it could also be used as a quality factor. Once the relationships between structural quality and optical anisotropy have been established, spectroscopic ellipsometry will presumably be the superior optical inspection technique for the rapid and non‐destructive wafer‐scale inspection of 2D materials for industrial mass production.

## 14 Enhancing Optical Signals for Fine Structural Characterization of 2D Materials

Because the atomically thin nature of 2D materials means that only a small portion of incident light interacts with them, the optical signals probed by optical inspection techniques are sometimes very weak, making it difficult to observe some of their structural characteristics. For example, Raman spectral studies of group‐III monochalcogenides have focused mainly on few‐layer crystals. Only a few reports have appeared of the Raman spectra of monolayer samples, due to their low signal‐to‐noise ratios and degradation under ambient environments.^[^ ^79^, ^244^ ^]^ On the other hand, the defect‐activated graphene D´ band (near 1600 cm^‐1^) is located at very close to the G band (near 1580 cm^‐1^);^[^ ^101^ ^]^ therefore, it usually appears as a small shoulder, making it particularly difficult to distinguish from the G band in a good‐quality sample. Similarly, the defect‐activated D_1_, D_1_´, D_2_, and D_2_´ bands of BP are located near the $A_{g}^{1}$ and $A_{g}^{2}$ modes^[^ ^245^ ^]^ and the photon energies of the trion emissions from TMDCs are merely 0.02 to 0.04 eV below their free exciton emissions; both of these sets of signals are, therefore, difficult to resolve from their neutral characteristics.^[^ ^177^ ^]^ Furthermore, although the edge chirality of BP, GeS, and GeSe can be identified using polarization‐dependent Raman spectroscopy, the samples are limited to thicker flakes.^[^ ^113^, ^114^ ^]^ The intensities of the Raman spectral characteristics for identifying the edge chirality of monolayers have been too small to be observed. Therefore, using optical inspection techniques to characterize the fine structural characteristics of 2D materials requires techniques to enhance their light–matter interactions.

Four strategies have been proposed to enhance the light–matter interactions of 2D materials.^[^ ^246^ ^]^ First, the 2D materials can be coupled to plasmonic metallic nanostructures, in which strong electromagnetic (EM) resonance occurs, so that an enhanced local electric field (E‐field) is generated. When the 2D materials are close to such plasmonic nanostructures, the light–matter interactions would be effectively enhanced and, hence, the absorption would increase. Nevertheless, EM resonance in a plasmonic nanostructure is limited to a narrow spectral regime; in addition, the enhanced local E‐field exists near the metallic nanostructure, and the strong light–matter interactions occur only when the 2D material is close to, or even contacting, the metallic structure. Any strong interactions between the metal and the 2D material will result in variations in their electrical or optical properties.^[^ ^246^ ^]^ Second, to overcome the disadvantages of using plasmonic nanostructures, the 2D materials can be integrated with waveguides or optical fibers made of dielectric materials (e.g., Si or SiO_2_). In this strategy, the materials that directly contact the 2D materials are those most commonly used in 2D material‐based devices; therefore, their interactions could be the same as those in real applications. When light propagates into the waveguides or optical fibers, the slight leakage of the waveguide mode would continuously interact with the attached 2D materials, such that the interaction length would be determined by the device length. Using this approach, near‐unity light absorption could be achieved, but such a configuration requires complicated fabrication processes, limiting its applicability.^[^ ^246^ ^]^ Third, the 2D materials could be integrated with an attenuated total reflection (ATR) configuration to achieve broadband absorption enhancement. When total internal reflection occurs, a lateral propagating evanescent wave appears at the reflection interface (where the 2D materials are located), increasing the interaction length between the incident light and the 2D material; hence, the amount of light absorbed could be greatly increased by using such an ATR configuration. Nevertheless, this strategy has a problem in that the enhancement is restricted to within a very specific angular range.^[^ ^246^ ^]^ Fourth, the 2D materials can be integrated with microcavity‐based structures comprising 2D materials sandwiched by two distributed Bragg reflectors (DBRs) to achieve a Fabry‐Perot cavity, or integrated with open nanocavity‐based structures (comprising a metal back reflector and a dielectric spacer) to enhance the light–matter interactions. The former can enhance the light absorption dramatically, but only at specific wavelengths and incident angles;^[^ ^246^ ^]^ the latter increases the light absorption moderately, but ultrabroadband and omnidirectional enhancement can be realized.^[^ ^247^ ^]^

In addition to enhancing the light absorption of 2D materials, attempts have also been made to enhance their Raman and PL signals through improved light–matter interactions, because Raman and PL processes involve excitation and, therefore, their signals are usually very weak. The most commonly applied method to increase the intensity of Raman and PL spectral signals of 2D materials is to integrate them with plasmonic nanostructures. Because of strong EM resonance in plasmonic nanostructures, the Raman scattering signals from 2D materials can be amplified significantly through so‐called “surface‐enhanced Raman scattering” (SERS)^[^ ^248^ ^]^ and their PL emissions can be enhanced greatly through “surface‐enhanced fluorescence” (SEF).^[^ ^249^ ^]^ Although the Raman and PL spectral signals of 2D materials can be enhanced using these methods, characterizing their original properties would be difficult because of the interactions between the plasmonic metal nanostructures and the 2D materials. The deposition of metallic nanoparticles directly onto the surface of 2D materials can result in defects; the transfer of 2D materials onto a metallic structure can induce strain or doping effects. Accordingly, their Raman or PL spectral characteristics can be seriously disturbed after integration with plasmonic nanostructures. Furthermore, strong EM resonance exists only in the gaps between adjacent metallic nanostructures, such that only a small area of the 2D material would experience this enhanced local E‐field and contribute most to the Raman or PL spectral signals. Thus, global properties could not be probed using plasmonic nanostructures. Accordingly, integration of 2D materials with plasmonic nanostructures is not an ideal method for enhancing their Raman and PL spectral signals.

**Figure 10:**
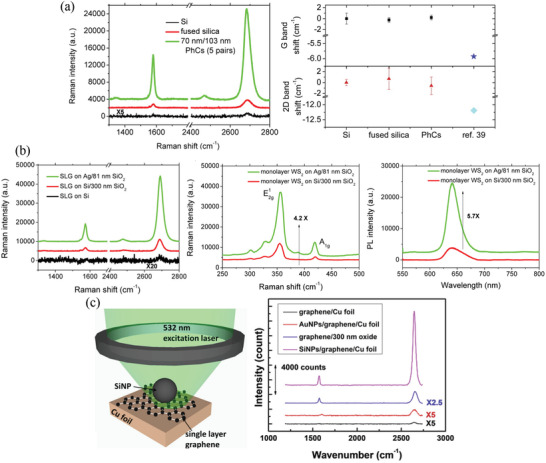
Techniques for enhancing optical signals from 2D materials. a) Left: Measured Raman spectra of monolayer graphene samples on silicon, fused silica, and 1D‐PhCs; right: Raman spectral peak shifts of monolayer graphene on various substrates (including a metallic substrate) relative to those on a silicon substrate. Reproduced with permission.^[^ ^251^ ^]^ Copyright 2015, American Chemical Society. b) Measured Raman spectra of monolayer graphene (left) and WS_2_ (middle) samples on various substrates; PL spectra of monolayer WS_2_ are also displayed (right). Reproduced with permission.^[^ ^252^ ^]^ Copyright 2016, American Chemical Society. c) Left: Schematic representation of the SiNP‐based method for enhancing optical signals directly from a graphene sample on Cu foil after CVD growth; right: measured Raman spectra of monolayer graphene samples on various substrates. Reproduced with permission.^[^ ^256^ ^]^ Copyright 2018, American Chemical Society.

Thin film‐based structures would be better alternative candidates for increasing the Raman and PL spectral signals of 2D materials, without disturbing their properties, thanks to pure interference‐enhanced effects.^[^ ^86^, ^87^, ^250^ ^]^ For example, a 1D photonic crystal (1D‐PhC) structure has been optimized to enhance the Raman spectral signals of monolayer and bilayer graphene while maintaining their band‐to‐band ratios and peak positions (**Figure** **10**a).^[^ ^251^ ^]^ The enhancements in both the graphene G and 2D bands were investigated systematically by tuning the optical properties of the 1D‐PhCs, thereby providing co‐enhanced Raman spectra of graphene. In addition, because the topmost layer of the 1D‐PhCs was SiO_2_, which interacts with graphene in the same manner as it would in graphene‐based devices, the structural properties of the graphene transferred onto the 1D‐PhCs could be modeled for real applications, with the peak positions of the Raman G and 2D bands being located at almost the same frequencies as those on silicon and fused silica (Figure 10a).^[^ ^251^ ^]^ Accordingly, using such 1D‐PhCs can indeed enhance the Raman spectra of graphene without any spectral distortion.

The interference‐based enhancement of the Raman and PL signals of 2D materials has been further optimized by using a nanocavity structure as the substrate, consisting of a silver back reflector and a SiO_2_ spacer (SiO_2_/Ag).^[^ ^252^ ^]^ As previously mentioned, such an open nanocavity structure enhances light–matter interactions in 2D materials in an ultra‐broadband manner; therefore, the enhancement factors of the graphene Raman G and 2D bands were higher than those obtained on the 1D‐PhCs, and the Raman spectra were non‐distorted (Figure 10b).^[^ ^252^ ^]^ Notably, the same nanocavity structure could also be used to enhance the PL spectra of monolayer WS_2_ (Figure 10b). Thus, nanocavity structures appear to be great platforms for precise investigations of the structural properties of 2D materials. Recently, the interference‐based enhancement method has been applied to characterize the pristine properties of monolayer graphene and MoS_2_ through investigations of their enhanced Raman and PL spectral signals on an air gap–based nanocavity.^[^ ^253^ ^]^ Furthermore, enhancements in the Raman and PL signals of vdW heterostructures, including InSe/MoS_2_,^[^ ^254^ ^]^ GaSe/InSe,^[^ ^254^ ^]^ and WSe_2_/MoS_2_,^[^ ^255^ ^]^ through interference effects have also been investigated systematically; while those studies focused on using SiO_2_/Si substrates, it is likely that such enhancements would be improved further by optimizing the nanocavity structures.

Metallic substrates [e.g., copper (Cu), nickel (Ni), gold (Au), tungsten (W)] are usually applied and regarded as catalysts for the CVD growth of high‐quality 2D materials.^[^ ^39^ ^]^ To monitor the fine structural properties of these CVD‐grown 2D materials and provide rapid feedback regarding the growth parameters, the most commonly used strategy is to transfer them onto a designed plasmonic or dielectric nanostructure (e.g., a standard 300 nm SiO_2_/Si substrate) and then measure their enhanced Raman and PL spectral signals. Nevertheless, the transfer procedure could have an unavoidable influence on such as‐grown 2D materials, disturbing their original characteristics. As a result, attempts have also been made to directly measure and analyze the optical signals (i.e., Raman/PL signals) of 2D materials on metallic substrates, without the need for transfer processes. When using Raman and PL spectroscopies to investigate the structural properties of CVD‐grown 2D materials on metallic substrates, enhancing techniques must always be applied because the Raman and PL spectral signals from 2D materials on metallic substrates are usually very weak.^[^ ^256^ ^]^ Several methods for enhancing the Raman and PL spectral signals have been proposed by depositing plasmonic nanoparticles onto 2D material/metallic substrates, but it has been difficult to retain the original properties, as mentioned above. To enhance the Raman spectral signals of 2D materials directly on metallic substrates, without any distortion of properties, Si nanoparticles (SiNPs) have been applied to enhance the Raman spectral signals of graphene on a Cu foil (Figure 10c).^[^ ^256^ ^]^ Because the SiNPs displayed strong magnetic dipole resonance and could effectively couple their EM field with the Cu substrate, high E‐field intensity was induced within graphene, thereby enhancing the Raman spectral signals significantly. The enhancement factors were even much greater than that on the standard 300‐nm SiO_2_/Si substrate. Notably, the SiNPs did not influence the peak positions or FWHMs of the Raman G and 2D bands of the as‐grown graphene on the Cu foil (Figure 10c), suggesting that they did not introduce any strain or doping effects on the graphene. Most importantly, the SiNPs were readily removed, making this method non‐destructive. That study established a very useful, practical method for directly characterizing as‐grown 2D materials on metallic substrates, without the need for transfer processes that could potentially change the original properties of the 2D materials.

## 15 Conclusions and Perspectives

Herein, we have reviewed several of the important structural properties of 2D materials and have discussed how they can be probed accurately with the use of optical inspection techniques. **Table** **1** summarizes the main characteristics of these optical inspection techniques. Based on their fundamental functions, they can be categorized into four groups. First, optical spectroscopies, spectroscopic ellipsometry, FTIR spectroscopy, and X‐ray scattering/diffraction/reflectivity are techniques that can be used to obtain broadband or omnidirectional optical spectra from various 2D materials. The detected spot size (diameter) in these optical inspection tools are usually large (from 100 µm to 1 cm), and the intensities of their light sources are relatively low; therefore, rapid characterization of 2D materials can be achieved when applying these techniques and, hence, they are suitable for inspecting the global uniformity—of great potential benefit for applications requiring the wafer‐scale inspection of 2D materials.^[^ ^241^, ^242^ ^]^ Nevertheless, the large spot size means that the spatial resolution when using these spectroscopic methods is quite low, such that the detailed structures of 2D materials cannot be inspected. Furthermore, the structural information from the 2D materials cannot be derived directly by analyzing their optical spectra; a fitting procedure, or establishment of the correlation between the structural parameters and optical signatures, remains necessary.^[^ ^240^, ^242^ ^]^ Second, Raman and PL spectroscopies are the powerful techniques that can provide abundant information regarding the fine structural characteristics of 2D materials. When the intensity of the light source and the sensitivity of the detectors are both sufficiently high, Raman and PL spectroscopic methods can provide moderate throughput. Notably, Raman and PL spectroscopies can be integrated with motorized stages, enabling mapping functions over larger areas, thereby increasing their throughput. Nevertheless, because Raman and PL spectroscopic methods require excitation to generate signals, the intensity of the incident laser light needs to be high, potentially inducing damage or increasing the local temperature of the samples through the effect of laser heating; as a result, characterization of the original properties of 2D materials can be difficult. To overcome this problem, enhancing techniques that do not disturb the Raman and PL spectral properties must be developed and applied when analyzing 2D materials.^[^ ^251^, ^252^ ^]^ Third, s‐SNOM, nano‐FTIR, and AFM‐IR spectroscopic methods are techniques based on AFM, such that they can achieve the highest spatial resolution. Although the high spatial resolution, determined by the size of AFM tip, is very helpful to obtain detailed structural properties in 2D materials, throughput is low. Interactions (or contact) between the AFM tip and the sample may have unwanted effects on the properties of the 2D sample. Fourth, single‐photon emission, TRPL, and pump‐probe spectroscopies provide time‐dependent optical signatures for characterizing 2D materials; information about the carrier dynamics, including carrier–photon, carrier–carrier, and carrier–phonon interactions, can be obtained. Nevertheless, these spectroscopic methods usually require complex instrumentation, and the pulsed laser light (rather than light from a CW laser) could possibly damage the samples, because the peak power is relatively high. Therefore, there are many pros and cons for each of the optical inspection techniques; a comprehensive understanding of their features, and which types of properties or characteristics they can reveal, will allow the researcher to adopt the appropriate tool for characterizing specific structural properties of 2D materials—our main goal with this Review. Table 1 elaborates the guidelines for choosing optical inspection techniques for characterizing 2D materials. Notably, the “check mark (✓)” in Table 1 means such structural properties or characteristics can be inspected and readily quantified; the “triangular mark (∆)” means that such properties would influence the signals obtained from the optical inspection techniques but their quantifications are not possible or still needed to be further investigated; the “blank cell” reveals the fact that no studies about using the optical inspection techniques to inspect such properties in 2D materials have been reported previously.

**Table 1:** Characteristics of optical inspection techniques for probing 2D materials

		OS^a)^	SE^b)^	Raman	PL	SPE	TRPL	PPS^c)^	s‐SNOM	FTIR	nano‐FTIR	AFM‐IR	XRS^d)^
Measured signals		T/R^e)^	Pol.‐R^f)^	RS^g)^	PL	PL	PL^j)^	T/R^e)^, ^j)^	IR light	T/R^e)^	IR light	CO^h)^	X‐ray
Light source		Lamp^k)^	Lamp^k)^	Laser	Laser	Laser	Pulsed laser	Pulsed laser	Laser	IR source^l)^	IR source^l)^	IR source^l)^	X‐ray
Spot size (diameter, µm)		10^3^–10^4^	10^2^–10^4^	1–20	1–20	1–20	1–20	300–10^3^	0.02–0.3	10^3^–10^4^	0.02–0.3	0.02–0.3	10^2^–10^3^
Throughput		Fast	Fast	Medium	Medium	Slow	Medium	Medium	Slow	Fast	Slow	Slow	Medium
Structural properties or characteristics can be inspected	R/T/A^i)^	✓								✓			
	Optical constants	✓	✓							✓			
	Layer #	✓	✓	✓	✓								✓
	Defect			✓	✓	✓	∆	∆					
	Strain			✓	✓								
	Doping			✓	✓								
	Carrier dynamics						✓	✓					
	Band structure				✓		∆						
	Polariton								✓				
	Functional group									✓	✓	✓	
	Stacking and twist			✓									
	Lattice spacing												✓
	Global properties	✓	✓							✓			

^a)^ OS, optical spectroscopy

^b)^ SE, spectroscopic ellipsometry

^c)^ PPS, pump‐probe spectroscopy

^d)^ XRS, X‐ray spectroscopy, including X‐ray scattering/diffraction/reflectivity

^e)^ T/R, transmitted/reflected light

^f)^ Pol.‐R, polarized reflected light

^g)^ RS, Raman scattering

^h)^ CO, cantilever oscillations

^i)^ R/T/A, reflectance/transmittance/absorptance spectra

^j)^ Time‐dependent measurement

^k)^ In broadband wavelength regime (from ultraviolet, visible, to near‐infrared)

^l)^ In broadband wavelength regime (from near‐infrared to mid‐infrared).

The vdW integration of 2D materials can extend their applicability to novel electronic, photonic, and optoelectronic devices; therefore, it can be crucial to monitor their optical and physical properties before and after integration. Although we have discussed several reports related to characterizing vdW integrations through optical inspection, the increasing number of discovered 2D materials means that there are thousands of possible combinations, increasing the complexity of investigations of vdW interactions. Moreover, studies of vdW integrations have mainly focused on analyses of Raman, PL, and ultrafast spectra. To fully understand the new physical properties of vdW interactions, it might be necessary to combine various optical inspection tools. As a result, considerable efforts will be required to develop new optical inspection for the vdW integrations of 2D materials.

For example, let us consider the band alignment of semiconducting 2D materials in vdW integrations; pump‐probe spectroscopy has mainly been used to examine their interlayer transitions and interlayer charge transfer,^[^ ^42^ ^]^ and PL spectroscopy has been used to determine their interlayer transition energies.^[^ ^229^ ^]^ Because the interlayer transition energies can correspond to specific absorption peaks, characterizing the absorptance spectra, obtained using optical spectroscopy, of vdW integrations should also provide information regarding their interlayer transitions. The broad‐bandwidth (from the visible to the mid‐IR) and large‐area detection provided by optical spectroscopy suggest that it should be easier to inspect the interlayer transition energies in vdW integrations without the need for precalculation of their electronic band structures. To date, however, only a few reports have described the absorptance spectra of vdW integrations determined using optical spectroscopy.^[^ ^42^ ^]^ Accordingly, there remains much room for further investigations of vdW integrations.

Regarding wafer‐scale inspection of 2D materials, spectroscopic ellipsometry appears to be the superior technique for rapidly and non‐destructively inspecting the structural quality of 2D materials, with the help of optical anisotropy as the quality factor; this concept has been verified for inspecting the global structural quality of graphene.^[^ ^241^, ^242^ ^]^ When applying spectroscopic ellipsometry to inspect the wafer‐scale growth of other 2D materials, the main challenge that remains will be establishing the relationships between the optical anisotropy and the structural quality. To address this problem, we suggest that studying the optical anisotropy of light absorption at the wavelengths of the characteristic absorption peaks (e.g., the exciton absorption or the absorption near the band edge) could be helpful for quantification of the structural quality. Furthermore, the substrate effect should also be taken into account to establish the most effective wafer‐scale inspection of 2D materials when applying spectroscopic ellipsometry.

In addition to the optical inspection techniques for characterizing 2D materials, we have also reviewed several enhancing techniques for increasing the light–matter interactions of 2D materials. Among these techniques, interference‐based enhancing methods appear to be most appropriate for 2D materials, because the intensity of their Raman and PL spectral signals can be increased effectively without distorting their spectra—a very helpful feature for fine structural characterization.^[^ ^251^, ^252^, ^253^ ^]^ Most importantly, the Raman and PL signals from 2D materials can be enhanced uniformly over the entire area, because the major enhancement mechanism of this method is an interference effect. Such homogeneous enhancement also provides the possibility of inspecting the global properties of 2D materials in conjunction with recently developed mapping techniques. Furthermore, we have described a technique involving the direct deposition of SiNPs for the in situ characterization of as‐grown graphene on Cu foil.^[^ ^256^ ^]^ EM coupling between the magnetic dipole resonance of the SiNPs and the Cu foil resulted in a local enhanced E‐field within the graphene, such that the Raman spectral signals were greatly enhanced without the need for transfer processes. With the help of interference‐ and SiNP‐based enhancing techniques, Raman and PL spectroscopies will become powerful tools for characterizing 2D materials.

During the development of 2D materials, the most important stage beyond vdW integrations and wafer‐scale growth will be their implementation in 2D material‐based devices. Several novel electrical and optical properties make 2D materials very suitable for application in electronic, photonic, and optoelectronic devices; not to mention their great potential for use in modern semiconductor devices [e.g., integrated circuits (IC)]. In the semiconductor industry, a well‐established optical inspection technique is optical critical dimension (OCD) measurement, which allows monitoring of the line widths and structural profiles of IC devices rapidly and non‐destructively by measuring diffracted or scattered light.^[^ ^257^ ^]^ As a result, OCD measurement remains a very useful technique for process control in semiconductor manufacturing. In the near future, as increasingly more 2D materials are integrated in ICs, their optical properties (e.g., optical constants and optical anisotropy) will presumably have to be taken into account in OCD measurements, to obtain correct information about the structural profiles of integrated 2D materials, especially for ICs having more complicated structures. Moreover, the Raman and PL characteristics of 2D materials integrated in ICs should also be considered during the microanalysis of device quality. Accordingly, these perspectives highlight the importance of comprehensively understanding the optical properties of 2D materials—as we have aimed to provide in this Review.

In conclusion, herein we have thoroughly reviewed the optical inspection techniques available for characterizing the structural properties of various 2D materials prepared through mechanical exfoliation, CVD growth, vdW integration, wafer‐scale growth, and beyond. We have also discussed various techniques for enhancing the optical signals from the 2D materials. We hope that this Review will lead to greater progress in developing novel 2D material‐based devices.

## Conflict of Interest

The authors declare no conflict of interest.
